# Proteomic comparison of osteoarthritic and reference human menisci using data-independent acquisition mass spectrometry

**DOI:** 10.1016/j.joca.2020.05.001

**Published:** 2020-08

**Authors:** E. Folkesson, A. Turkiewicz, N. Ali, M. Rydén, H.V. Hughes, J. Tjörnstrand, P. Önnerfjord, M. Englund

**Affiliations:** † Lund University, Faculty of Medicine, Department of Clinical Sciences Lund, Orthopaedics, Clinical Epidemiology Unit, Lund, Sweden; ‡ Lund University, Faculty of Medicine, Department of Clinical Sciences Lund, Rheumatology and Molecular Skeletal Biology, Lund, Sweden; § Department of Orthopaedics, Skåne University Hospital, Lund, Sweden; ‖ Clinical Epidemiology Research and Training Unit, Boston University School of Medicine, Boston, MA, USA

**Keywords:** Osteoarthritis, Meniscus, Mass spectrometry, Extracellular matrix

## Abstract

**Objective:**

Recent research in knee osteoarthritis (OA) highlights the role of the meniscus in OA pathology. Our aim was to compare the proteomes of medial and lateral menisci from end-stage medial compartment knee OA patients, with reference menisci from knee-healthy deceased donors, using mass spectrometry.

**Design:**

Tissue plugs of Ø3 mm were obtained from the posterior horns of the lateral and medial menisci from one knee of 10 knee-healthy deceased donors and 10 patients undergoing knee replacement. Proteins were extracted and prepared for mass spectrometric analysis. Statistical analysis was conducted on abundance data that was log_2_-transformed, using a linear mixed effects model and evaluated using pathway analysis.

**Results:**

We identified a total of 835 proteins in all samples, of which 331 were included in the statistical analysis. The largest differences could be seen between the medial menisci from OA patients and references, with most proteins showing higher intensities in the medial menisci from OA patients. Several matrix proteins, e.g., matrix metalloproteinase 3 (MMP3) (4.3 times higher values [95%CI 1.8, 10.6]), TIMP1 (3.5 [1.4, 8.5]), asporin (4.1 [1.7, 10.0]) and versican (4.4 [1.8, 10.9]), all showed higher abundance in medial menisci from OA patients compared to medial reference menisci. OA medial menisci also showed increased activation of several pathways involved in inflammation.

**Conclusion:**

An increase in protein abundance for proteins such as MMP and TIMP1 in the medial menisci from OA patients suggests simultaneous activation of both catabolic and anabolic processes that warrants further attention.

## Introduction

Osteoarthritis (OA) is a chronic joint disease traditionally characterized by loss of articular cartilage and changes in the underlying bone. However, recent research in knee OA has highlighted an important role for the meniscus in OA etiology and pathogenesis^1^, ^2^, ^3^. The menisci are two wedge shaped semi-circular fibrocartilage structures interposed between the femoral and tibial condyles in the knee, and their main function is load transmission^2^^,^^3^. Studies have reported that damage to the meniscus, which can occur due to acute knee trauma or as a result of gradual degenerative changes, is strongly associated with increased risk for knee OA^4^, ^5^, ^6^. Slow degradation of meniscal tissue has been suggested to occur in early stages of OA, however little is known of the molecular processes^7^^,^^8^. There is also very limited understanding of the molecular composition of the meniscus. Thus, more knowledge is needed about the meniscus, both in health and OA.

Mass spectrometry (MS) coupled with liquid chromatography (LC) is one of the most powerful methods to analyze protein content in complex samples. With non-targeted MS, it is possible to identify thousands of proteins in a single analysis^9^^,^^10^. In a previous study we investigated the proteomic differences between three zones (peripheral, middle and inner) of normal human menisci, as a first step to gain new knowledge about the human meniscus^11^. In the present study, our aim was to gain further insight into the role of the meniscus in OA pathogenesis, by comparing tissue plugs from the posterior horn of normal human menisci and menisci taken from medial compartment knee OA patients, using a global MS approach.

## Material and methods

### Materials

N-Ethylmaleimide, 6-aminocaproic acid, benzamidine hydrochloride hydrate, dithiothreitol (DTT), iodoacetamide, ammonium bicarbonate (AMBIC), formic acid, and HPLC grade acetonitrile and Solvents A (0.1% formic acid) and B (0.1% formic acid in acetonitrile) for liquid chromatography-mass spectrometry (LC-MS) were from Sigma–Aldrich (St. Louis MO, USA). Guanidine hydrochloride (GdnHCl) and anhydrous sodium acetate (NaAc) were from Merck (Darmstadt, Germany). Trypsin Gold (MS grade) was purchased from Promega (Madison WI, USA). The Pierce Quantitative Colorimetric Peptide Assay and SOLAμ™ Solid Phase Extraction (SPE) HRP (horse radish peroxidase) 2mg/1 ml 96-well plates were from Thermo Fisher Scientific (Rockford IL, USA), and Nanosep® 30K Omega Centrifugal Devices were from Pall Life Sciences (Ann Arbor MI, USA).

### Selection of meniscus samples

We sampled meniscal tissue from a previously described biobank of human knee tissues at Skåne University Hospital, Lund, Sweden^12^^,^^13^, following approval by the regional ethics committee in Lund (2015/39 and 2016/865).

**Fig. 1:**
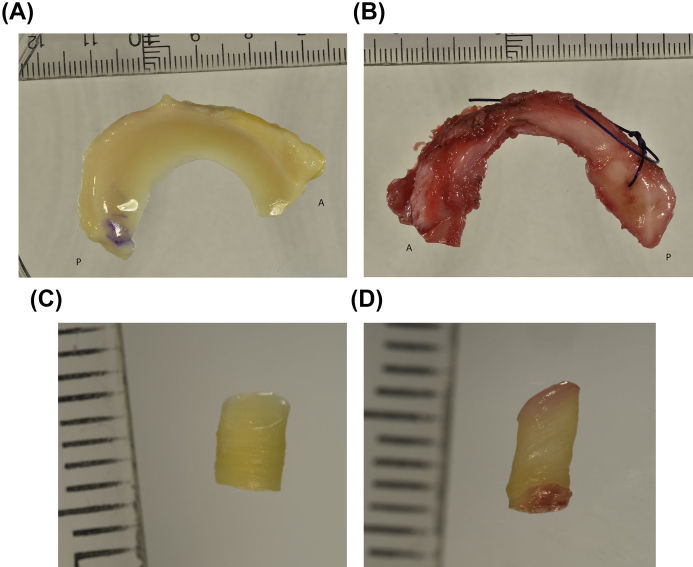
Representative images of A) reference meniscus (from a right knee), B) OA meniscus (from a left knee), C) plug from a reference meniscus and D) plug from an OA meniscus. In panels A and B, the annotations “A” and “P” refer to the anterior and posterior ends of the meniscus, respectively.

Reference menisci: From the biobank, we selected both medial and lateral menisci from the right knees of 10 adult, deceased donors (five women, five men) (convenience sample) with no known diagnoses of rheumatoid arthritis or knee OA. All menisci were obtained within 48 h post-mortem, and the specimens were frozen at −80°C within 2 h of extraction. To be eligible as references, the donor menisci were required to be macroscopically intact (some minor calcifications were allowed) [Fig. 1(A)]. Further, we also inspected the femoral cartilage (the load bearing region) from the medial compartment of the same donors (also in the biobank) and required the cartilage to be macroscopically intact.

The donor menisci selected for this study will hereafter be called reference menisci, with samples from the medial and lateral menisci referred to as medial^ref^ and lateral^ref^, respectively.

OA menisci: Medial and lateral menisci from 10 patients (five women, five men) (convenience sample) with medial compartment knee OA were selected from the biobank. The menisci were retrieved during total knee replacement (TKR) surgery and frozen at −80°C within 2 h after surgery at Trelleborg Hospital and later transported to Lund on dry ice for further storage at −80°C in the biobank. The surgeon classified the knee joint cartilage of all patients according to the Outerbridge classification system during surgery. In order to be classified as medial compartment knee OA, the Outerbridge grade was required to be IV (exposed bone) in the medial compartment and 0 (normal) or I (softening of cartilage) in the lateral compartment^14^. We further required the medial menisci from these patients to have at least two thirds of the substance of the posterior horn remaining (the inner one third was typically missing due to degeneration) [Fig. 1(B)]. The lateral OA menisci were usually intact at the macroscopic level.

Samples from the medial and lateral menisci from the medial compartment knee OA patients selected for this study will hereafter be referred to as medial^OA^ and lateral^OA^, respectively.

### Preparation of meniscal tissue for MS analysis

**Fig. 2:**
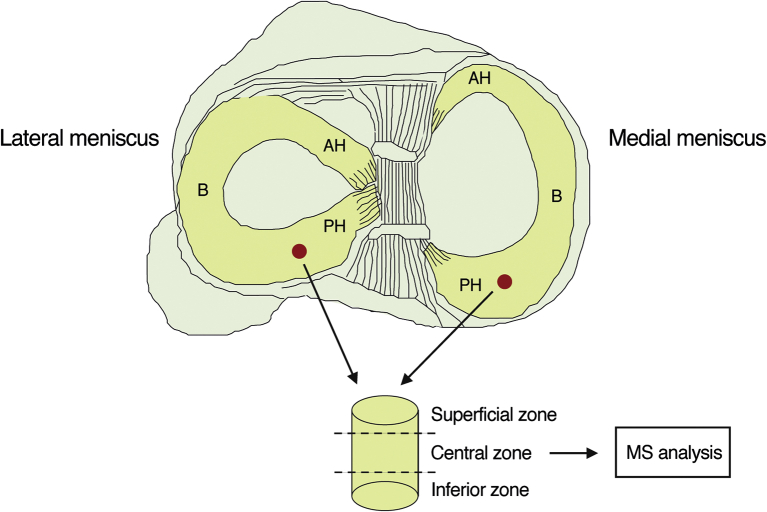
*Top:* Schematic figure showing the location of the collected meniscal plugs from lateral and medial menisci. AH: anterior horn, B: body, PH: posterior horn. *Bottom:* Of the different zones of the plugs, only the central zone was used for MS analysis.

Menisci were thawed in phosphate-buffered saline (PBS). To prepare the tissue samples for MS analysis, a hole (3 mm in diameter) was punched vertically through the central part of the posterior horn (approximately 3–5 mm from the capsular insertion, i.e., in the thicker peripheral part of the meniscus) [Fig. 1, Fig. 2]. The superficial and inferior ends (1/4 each) of the plugs (*n* = 40) were cut off and stored at −80°C (Fig. 2). The central parts of the plugs were pulverized in liquid nitrogen using a pestle and mortal technique, after which the pulverized tissue was weighed. Proteins were extracted using 15 volumes (15 μL buffer/mg dry powder) of chaotropic buffer (4 M GdnHCl, 50 mM NaAc, 100 mM 6-aminocaproic acid, 5 mM benzamidine, 5 mM N-ethylmaleimide, pH 5.8), incubated for 24 h on an orbital shaker at +4°C, and centrifuged at 13,200×*g* at +4°C for 30 min. The resulting supernatant was used for further analysis. This process was repeated twice on the extraction pellet remaining after the centrifugation.

Equal volumes from the three sequential extracts were pooled, resulting in 180 μL of extract per sample. For three of the samples it was only possible to take 20 μL from each extraction round due to sample losses during preparation. Pooled samples were reduced using 4 mM DTT, with shaking at +56°C for 30 min. Extracts were alkylated using 16 mM iodoacetamide for 1 h at room temperature in the dark. In order to remove residual salts, extracts were precipitated with 9 volumes of ethanol incubated for 4 h at 20°C, after which the precipitate was dried in a SpeedVac and suspended in 100 μL of 0.1 M AMBIC buffer, pH 8.5. All samples were digested using 2 μg Trypsin Gold, by incubating on a shaker at +37°C for approximately 16 h. Peptide concentrations of the digests were determined using the Pierce Quantitative Colorimetric Peptide Assay according to the manufacturer's instructions. Samples (30 μg) were diluted to 200 μL in 50 mM AMBIC buffer with 0.5 M sodium chloride (to minimize ionic interactions). In order to remove peptides with glycosaminoglycan (GAG) chains from the samples, they were centrifuged through Nanosep® 30K Omega Centrifugal Devices. Samples were subsequently desalted using Thermo Scientific™ SOLAμ™ SPE plates and eluted using 50% acetonitrile in 0.1% formic acid.

At the end of sample preparation, from the original 40 samples selected for analysis (i.e., 10 samples each of medial^OA^, lateral^OA^, medial^ref^, and lateral^ref^), seven had to be omitted due to sample preparation problems and lack of sufficient material. This resulted in *n* = 9 medial^OA^, *n* = 7 lateral^OA^, *n* = 8 medial^ref^, and *n* = 9 lateral^ref^ samples for further MS analysis.

### Mass spectrometry analysis

Digested samples were analyzed using a Q-Exactive™ HF-X quadrupole Orbitrap benchtop mass spectrometer (Thermo Fisher Scientific). Equal amounts of the protein digests were injected into an Easy nano-LC 1000 HPLC system (Thermo Fisher Scientific) equipped with an Acclaim PepMap® 100 nanoViper pre-column (Thermo Scientific, C18, 3 μm particles, 75 μm i.d. 2 cm long) and a PepMap® RSLC C18 analytical column (Thermo Scientific, C18, 2 μm particles, 75 μm i.d. 25 cm long) (RSLC: rapid separation liquid chromatography). On-line reversed-phase separation was performed using a flow rate of 300 nL/min. A binary gradient of 125 min was used, starting with a 5 min increase from 5% B to 7% B, then increasing to 20% B over 85 min, further increasing to 30% B over 20 min, and with a final 5 min increase to 90% B, after which it ended with 90% B isocratic for 10 min. The system was controlled by Xcalibur™ Software (Thermo Fisher Scientific), and blank runs were injected between every sample to avoid cross-contamination. A heated ion transfer setting of 280°C was used for desolvation, together with a spray voltage of +1850 V.

### Data-independent acquisition (DIA)

We ran all samples in Data-independent acquisition (DIA) mode using a blocked randomization sequence. Each block contained one sample from each group, and the medial and lateral samples from each reference subject and OA patient were run in the same block. Two randomly selected samples from each group were selected to be run in duplicates to perform reliability assessment. All duplicates were run in a randomized order within the same block. The first replicate of each duplicate was used for statistical analysis.

For the MS settings, the full MS scan (350–1650 *m*/*z*) was set to a resolution of 120,000, with 3.0 × 10^6^ automatic gain control (AGC), and 100 ms maximum ion injection time. This was followed by DIA collision-induced dissociation MS2 scans at a resolution of 45,000, with 3.0 × 10^6^ AGC, and an automatic maximum ion injection time. A loop count of 26 was used with variable isolation windows (Table S1). To check for instrument performance, a pooled sample was injected every 10th injection.

### Data-dependent acquisition (DDA)

Fourteen samples representing all sample groups (i.e., medial^ref^, lateral^ref^, medial^OA^ and lateral^OA^) were randomly selected to be run in Data dependent acquisition (DDA) mode in order to make a combined spectral library using both DDA and DIA data for the quantitative data analysis. Eight of the DDA runs were run before the DIA runs, and six were run after. The run order was randomized. For the DDA analysis, the full MS scan (375–1400 *m*/*z*) was set to a resolution of 120,000, with 3.0 × 10^6^ AGC, and 50 ms maximum ion injection time. This was followed by collision-induced dissociation MS2 scans with a resolution of 15,000, AGC of 1.0 × 10^5^ and maximum injection time of 30 ms.

### Data analysis

A total of 61 MS runs (14 DDA and 47 DIA) of the meniscal samples were converted to HTRMS format using HTRMS Converter (Biognosys AG, Switzerland) in order to decrease data analysis search time. A spectral library was created in Spectronaut X™ Pulsar (version 12.0.20491.15, Biognosys AG) using all DIA and DDA runs. Default settings (BGS factory settings) were used with additional modifications: cysteine carbamidomethylation as a fixed modification, and deamination, pyro-glutamic acid (N-term Glu to pyroglutamic acid), methionine oxidation, hydroxyproline and acetylation as variable modifications. The human protein database (20190416_UP152602_n20415) was used as the background proteome. A subsequent protein search was conducted in Spectronaut™ Pulsar using the recently created spectral library and the same human database as background proteome. Default settings were used for the search. Precursor quantitation was performed at MS2 level, and area under the curve was used as quantitation type. The top three peptides (proteotypic) for each protein were averaged to calculate protein abundance^15^.

### Statistical analysis

In the statistical analysis, we included proteins with a maximum of one missing value per sample group (medial^ref^, lateral^ref^, medial^OA^ and lateral^OA^), which provided a total of 331 proteins for analysis. All intensities were log_2_-transformed before the analysis. Thus, the presented estimates in figures and tables are differences between mean log_2_ intensities between groups, whose inverse 2^x^ (where x is the estimate), provides the ratio of the geometric means (often referred to as fold-changes, which are the estimates presented in the text). We used a linear mixed-effects model, with the sample group, protein type, and their interactions as independent variables, and transformed intensities as the outcome. Subject identifiers were included as random effects to account for correlation between measurements from the same individual. We adjusted the model for the potential confounders age and body mass index (BMI). This adjustment has very limited impact on the within person comparisons (i.e., medial^OA^ vs lateral^OA^ and medial^ref^ vs lateral^ref^), which are adjusted for person-invariant confounders by design. Using one multilevel model for simultaneous analysis of all included proteins results in more efficient estimation and better control of so-called type I error^16^. Presented estimates are with 95% confidence intervals (CI).

### Functional enrichment analysis

A functional enrichment analysis was performed in Ingenuity® Pathway Analysis (IPA®, QIAGEN Bioinformatics, Fall release, 2019) to determine if any pathways were overrepresented in our data. In this analysis, we focused on the comparison between medial^OA^ and medial^ref^ menisci, and only proteins with a log_2_ intensity difference of over 1.2 or under −1.2 (i.e., fold-changes over 2.3 or under 0.4) were included. IPA® also evaluates the proteins based on upstream regulators and calculates a z-score; positive for regulators with activating capacity and negative for those with inhibitory capacity. The z-score is a statistical measure of the match between the expected direction of the relationship and the observed protein expression, which is used to determine how strong the overrepresentation is. Default settings were used, with species set to human and all identified proteins in this study used as background proteome. Five proteins (hemoglobin subunit alpha (P69905), histone H4 (P62805), and immunoglobulin chains alpha-2 heavy (P0DOX2), gamma-1 heavy (P0DOX5) and kappa light (P0DOX7)) could not be mapped in IPA and therefore had to be excluded from the analysis.

## Results

The 10 reference subjects had a median age of 51 years (range 18–77) and median BMI of 26.2 (range 16.4–42.4) (Table I). The nine OA patients (after exclusion of one patient due to sample loss) had a median age of 61 years (range 50–75) and median BMI of 28.7 (range 26.5–37.4) (Table I).

**Table I:** Characteristics of the study sample

Subject number	Group	Sex	Age (years)∗	BMI (kg/m^2^)	Knee side	Medial (M)/Lateral (L)†
1	Reference	Female	18	16.4	Right	M
2	Reference	Female	32	22.8	Right	M/L
3	Reference	Female	61	23.3	Right	M
4	Reference	Female	74	25.5	Right	M/L
5	Reference	Female	77	22.3	Right	M/L
6	Reference	Male	49	33.0	Right	L
7	Reference	Male	50	34.2	Right	M/L
8	Reference	Male	52	26.8	Right	M/L
9	Reference	Male	58	33.2	Right	M/L
10	Reference	Male	43	42.4	Right	M/L
References: Mean (SD)	–	–	**51.4 (17.9)**	**28.0 (7.6)**	–	
11	OA	Female	50	37.4	Left	M/L
12	OA	Female	53	22.5	Left	M
13	OA	Female	60	29.4	Left	M/L
14	OA	Female	61	30.5	Right	M/L
15	OA	Female	61	25.9	Right	M
16	OA	Male	61	30.4	Right	M/L
17	OA	Male	65	27.5	Right	M/L
18	OA	Male	72	28.7	Left	M/L
19	OA	Male	75	28.7	Right	M/L
OA patients: Mean (SD)	–	–	**62 (8.0)**	**29.0 (4.0)**	–	
All subjects: Mean (SD)	–	–	**56.4 (14.7)**	**28.5 (6.0)**	–	

∗ At time of death (references) or at the time of surgery (OA patients).

† Menisci included in the final MS analysis.

In total, we identified a total of 835 proteins. However, in order to be included in the statistical analysis, proteins were allowed to have a maximum of one missing value per sample group, which resulted in 331 proteins remaining. Of these, 217 proteins had no missing values at all. There were on average more missing values in the reference menisci compared to the OA menisci (Supplementary Table S2).

**Fig. 3:**
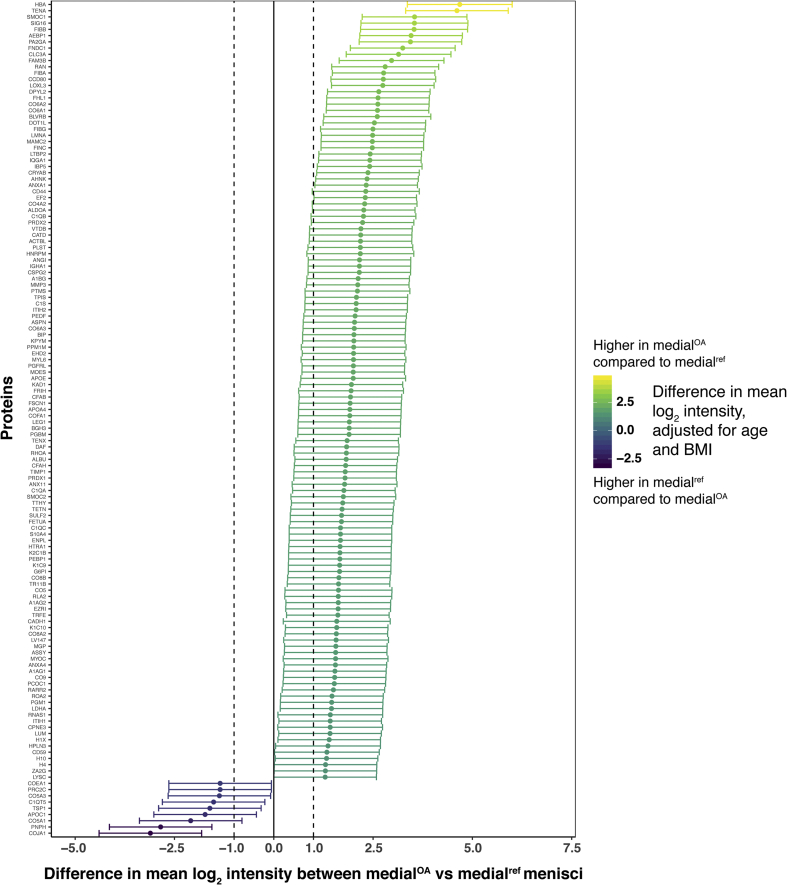

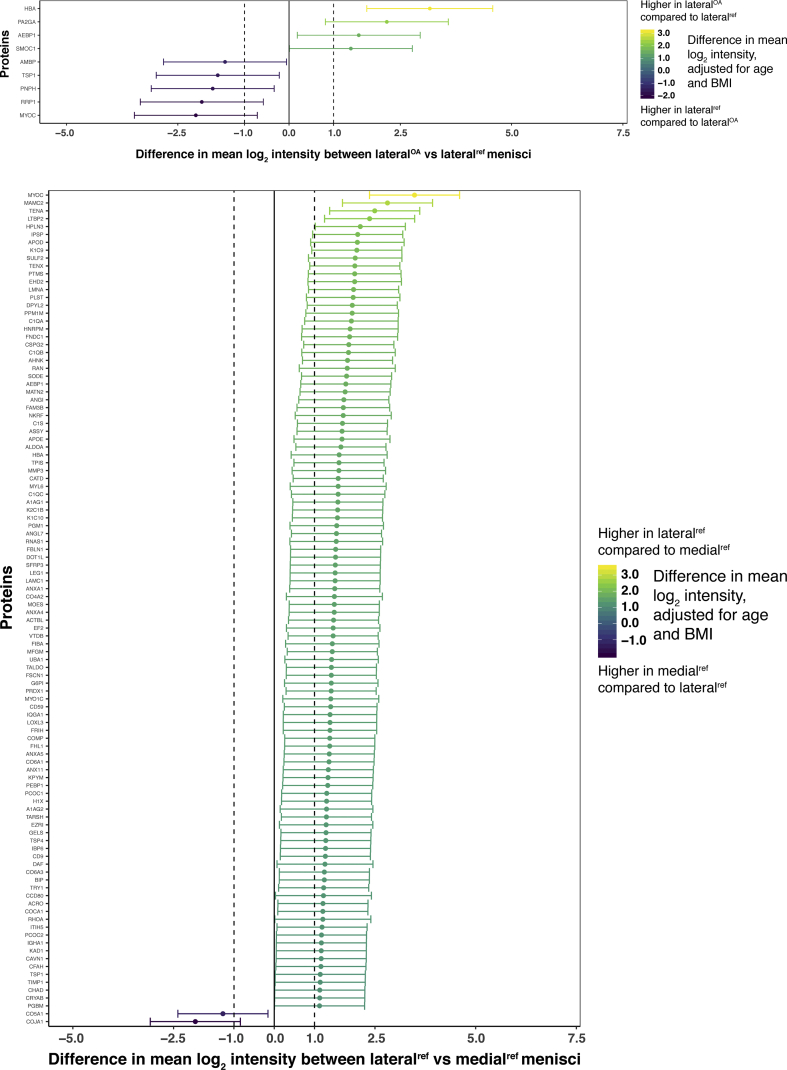

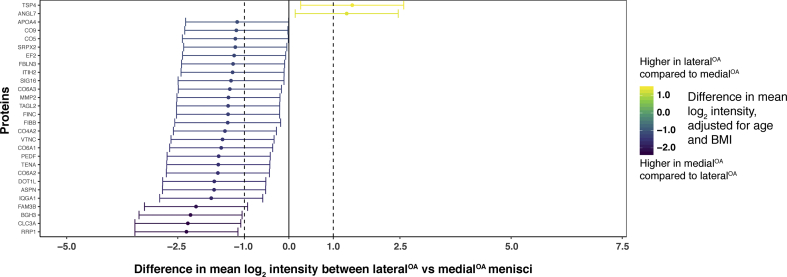
Visual representation of differentially expressed proteins (y-axis) in A) medial^OA^ vs medial^ref^ menisci, B) lateral^ref^ vs medial^ref^ menisci, C) lateral^OA^ vs medial^OA^ menisci and D) lateral^OA^ vs lateral^ref^ menisci. Log_2_-transformed mean intensity differences are displayed as point estimates with 95% confidence intervals as error bars (x-axis). The color of the point estimates and error bars represents the difference in log_2_ mean intensity between the groups, according to the scale bar on the right.

The biggest differences in protein intensities were found between the medial^OA^ and medial^ref^ menisci, with a majority of proteins showing a higher mean log_2_ intensity in the medial^OA^ group [Fig. 3(A)]. In contrast, very few differences were found between the lateral^OA^ and lateral^ref^ menisci [Fig. 3(B)]. In within-person comparisons of lateral and medial menisci from the same knees, lateral^ref^ generally had higher log_2_ protein intensities than medial^ref^ among reference menisci [Fig. 3(C)], whereas most proteins had similar intensities in lateral^OA^ and medial^OA^ menisci [Fig. 3(D)]. All comparisons of proteins included in the statistical analysis is detailed in Table S3 and Figs. S1–4.

The protein with the largest fold-change between OA and reference menisci (both medial and lateral) was hemoglobin subunit alpha (HBA), with a higher mean intensity in the OA menisci (fold-change 25.63 with 95% CI [10.27, 64.0] for the medial menisci and 8.94 [3.36, 23.92] for the lateral) [Fig. 3(A) and (D)]. The protein with the largest fold-change between medial^OA^ and medial^ref^ menisci, but with a higher mean intensity in the medial^ref^ menisci (0.12 [0.05, 0.28]) [Fig. 3(A)], as well as between lateral^ref^ and medial^ref^ menisci (0.26 [0.12, 0.56]) [Fig. 3(C)], was collagen alpha-1(XIX) chain (COJA1). Myocilin (MYOC) was the protein with the largest fold-change between lateral^ref^ menisci and lateral^OA^ menisci (0.23 [0.09, 0.61]) (Fig. 3(B), as well as between medial^ref^ menisci and lateral^ref^ menisci (11.16 [5.13, 24.25]) [Fig. 3(C)]. Finally, the protein with the largest fold-change between lateral^OA^ menisci and medial^OA^ menisci was thrombospondin-4 (TSP4) (2.69 [1.21, 6.02]), whereas it was ribosomal RNA processing protein 1 homolog A (RRP1) in medial^OA^ menisci compared to lateral^OA^ menisci (0.20 [0.09, 0.45]) [Fig. 3(D)].

Several proteoglycans were identified in this study, including 10 major small leucine-rich proteoglycans (SLRPs). In comparing medial^OA^ menisci to medial^ref^ menisci, all but one of the SLRPs had a higher intensity in the medial^OA^ menisci. The two SLRPs with the largest fold-changes were asporin (4.08 [1.67, 9.99]) and lumican (2.68 [1.09, 6.54]) [Fig. 3(A)]. Among the SLRPs, decorin was the only protein with a lower mean intensity in the medial^OA^ menisci compared to the medial^ref^ menisci, with a fold-change of 0.51 [0.21, 1.25]) (Table S3). All SLRPs had similar intensities in lateral^OA^ and lateral^ref^ menisci (Table S3). In within-person comparisons between lateral and medial menisci, only asporin was differentially expressed between lateral^OA^ and medial^OA^ menisci (increased in medial^OA^ with a fold-change of 0.31 [0.14, 0.70]), whereas it had a fold-change of 1.89 [0.87, 4.08] between the medial^ref^ and lateral^ref^ menisci [Fig. 3(C) and (D)], and chondroadherin was the only differentially expressed SLRP between lateral^ref^ and medial^ref^ menisci (increased in lateral^ref^ with a fold-change of 2.18 [1.0, 4.76]), with a similar fold-change between the lateral^OA^ menisci and medial^OA^ menisci (1.41 [0.63, 3.16]) [Fig. 3(C) and (D)].

The other proteoglycans identified in this study, aggrecan, versican, perlecan, and collagen alpha-1 (XVIII) chain, were all increased in medial^OA^ compared to medial^ref^ menisci, where versican and perlecan had the largest fold-changes. Both were increased in medial^OA^ compared to medial^ref^ menisci, with fold-changes of 4.44 [1.82, 10.85] and 3.73 [1.53, 9.13], respectively [Fig. 3(A)]. The same two proteoglycans were also elevated in lateral^ref^ vs medial^ref^ menisci from the same knees, with fold-changes of 3.61 [1.66, 7.84] and 2.17 [1.0, 4.72], respectively [Fig. 3(C)]. Both of these proteoglycans had similar intensities in lateral^OA^ compared to lateral^ref^ menisci (versican: 0.82 [0.31, 2.13], perlecan: 1.07 [0.41, 2.79]) and lateral^OA^ compared to medial^OA^ menisci (versican: 0.66 [0.30, 1.48], perlecan: 0.63 [0.28, 1.40]) (Table S3).

**Fig. 4:**
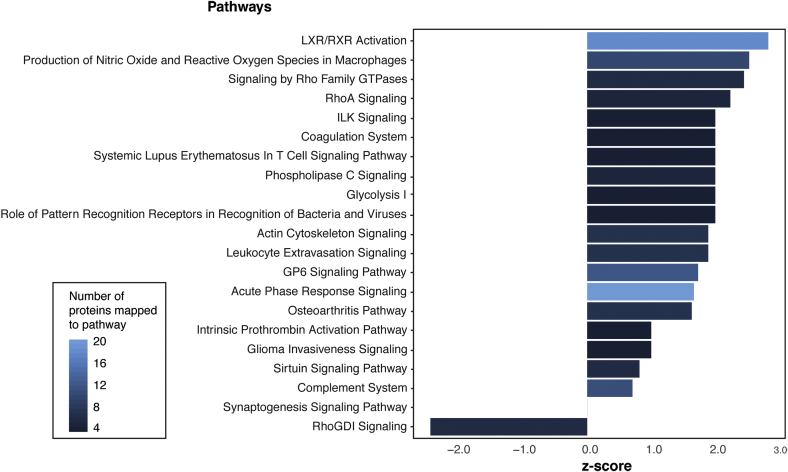
Results from the pathway analysis on the proteins differing between the medial^OA^ and medial^ref^ menisci, which was performed in IPA®. Bar plot displaying the most increased or decreased pathways mapped to the proteins. Z-score is a statistical measure of the match between the expected direction of the relationship and the observed protein expression and a higher z-score corresponds to a stronger overrepresentation. The color of the bars represents the number of proteins that could be mapped to that specific pathway.

Furthermore, we identified several canonical pathways that were enriched in the comparison between medial^OA^ and medial^ref^ menisci (Fig. 4 and Table S4). All pathways were increased in medial^OA^ menisci except RhoGDI signaling (z-score −2.45) which was decreased. Liver X receptors (LXR)/retinoid X receptors (RXR) activation had the highest z-score among the pathways increased in medialOA menisci (z-score 2.83). In addition, many of the upregulated pathways were associated with inflammation.

## Discussion

We compared the proteome of human meniscal posterior horns from knee-healthy references and end-stage medial compartment knee OA patients. Medial menisci from OA patients compared to references shows clear differences in protein expression, consistent with the disease pathology.

Several proteoglycans showed higher intensity in medial^OA^ menisci compared to the medial^ref^ menisci. It might seem counter-intuitive to see an increase in proteoglycans in osteoarthritic tissue. However, in a previous study where we investigated the proteoglycan content in the same reference and OA menisci as in this study, by staining meniscus sections with Safranin-O-Fast Green staining, we observed increased staining with increasing degeneration of the menisci^13^. This finding is supported by others, and have been hypothesized to be an attempt at repair^17^^,^^18^.

Interestingly, HBA was the protein with the largest difference between OA and reference menisci (both medial and lateral), with a higher mean intensity in the OA menisci. In addition, several high abundant human plasma proteins also had higher mean intensities in the medial^OA^ menisci. This might be due to the fact that there was intra-articular bleeding in the OA patients' knees during TKR surgery, with collected menisci contaminated with blood. However, before dissection, every meniscus was rinsed with PBS. The OA menisci should therefore, in theory, have been as free of blood on the surface as the reference menisci. Instead, since menisci are vascularized^19^, another possible explanation might be that there are internal hemorrhages within the menisci of OA patients, caused by tissue damage and degeneration.

The glycoprotein myocilin showed the highest mean intensity in lateral^ref^ menisci compared to lateral^OA^ menisci. We previously identified myocilin in both articular cartilage and meniscus, found to be more abundant in the meniscus than articular cartilage^20^. In addition, a previous proteomic study reported higher expression of myocilin in synovial fluid from healthy controls compared to individuals with meniscal injuries or pathologies^21^, which is consistent with our findings. This indicates that myocilin might be involved in pathological processes of the meniscus, having a possible role in the pathogenesis of OA.

In the pathway analysis, several pathways could be mapped to the proteins that differed between the medial^OA^ and medial^ref^ menisci. The pathway with the highest z-score was LXR/RXR activation, which was increased in the medial^OA^ menisci. As members of the nuclear receptor superfamily, LXR and RXR can form heterodimers with each other, and function as transcription factors that regulate many different physiological processes^22^^,^^23^. Interestingly, studies have reported the involvement or dysregulation of LXR/RXR pathways in OA tissue^24^, ^25^, ^26^, ^27^. One of these studies reported that treatment of murine articular chondrocytes with RXR agonists decreased the expression of aggrecan and fibrillin-2 genes and increased the expression of MMP13 genes, changes associated with tissue degradation^25^. Interestingly, the same study also reported an opposite effect for treatment with LXR agonists, which decreased the expression of MMP13 and MMP2, which are genes associated with cartilage breakdown, indicating that LXR activation might act protectively on articular cartilage. We also observed a simultaneous increase of both the proteinase MMP3, which induces breakdown of cartilage, as well as its inhibitor TIMP1, in the medial^OA^ menisci compared to the medial^ref^ menisci [Fig. 3(A)]. Taken together, these results indicate that there is an on-going degradation process in the OA menisci, but also a simultaneous attempt to stop the degradation.

Furthermore, one of the most striking results from the pathway analysis was the activation of several pathway associated with inflammation, such as production of nitric oxide and reactive oxygen species in macrophages, acute phase response signaling and complement system. Traditionally, OA has not been described as an inflammatory disease, however, it has been become widely accepted that inflammation plays a role in OA, with higher levels of inflammatory cytokines in synovial fluid and serum of OA patients^28^, ^29^, ^30^, ^31^. In addition, a study comparing the transcriptome of OA and non-OAOA menisci, reported an increase of genes involved in inflammation, in the OA menisci, supporting our findings.

Several of the differentially expressed proteins were collagens. However, this might not be a result of biological differences, but rather a result of the protein extraction method used in this study, which is based on guanidine hydrochloride. This method is not optimal for collagen extraction and depending on the extent of collagen cross-linking or subtypes in different samples, the results may vary^32^. It might therefore be premature to make any interpretation about collagens from our study, without further validation.

Finally, we would like to highlight some important limitations in our study. First, due to sample preparation issues, seven samples had to be omitted from the original 40 samples planned for analysis. However, since we consider this to be a pilot study, this was deemed acceptable and we acknowledge the need for future replication studies. Second, even though we use macroscopically intact menisci from donors without known OA, we recognize that there are variations among the donors in the extent of tissue degradation, which is why we prefer to refer to them as reference menisci, rather than “healthy” or “normal”. Further, unfortunately, the cause to the patients’ knee OA was not known, but is also in practice hard to ascertain as it is often a combination of hereditary and environmental factors. Furthermore, we observed a large amount of missing values in our dataset, and decided to use a very stringent approach of excluding proteins with more than one missing value per sample group, which decreased the number of proteins for analysis from 835 to 331. There are several possible reasons as to why there were so many missing values. One reason is that some proteins were of low abundance and could not be quantified at all, or only in some of the samples. However, several proteins exhibited interesting expression patterns, having many missing values in the reference samples and few in the OA samples (Table S2). This, together with the fact that the medial menisci appeared to have more missing values than the lateral menisci, indicates that there may be a biological explanation to the missing data. It would therefore be interesting to, in future studies, look more closely at the proteins with interesting patterns of missing data. Moreover, peptides with GAG chains were removed in order to avoid introducing GAG chains to the LC-MS system as they are detrimental for its performance. This could result in a potential loss of proteins via interactions with GAG chains (at peptide level), however this is circumvented by performing this step with high salt levels and thus these proteins should be detected as usual. Finally, there is unfortunately no material left for validation of our findings by other methods.

In conclusion, it appears that the largest differences in the meniscal proteome, as expected, could be seen between medial^OA^ and medial^ref^ menisci. The majority of the proteins that differed, e.g., MMP3, TIMP1 and several proteoglycans, had higher intensities in the medial^OA^ menisci, indicating that there is an overall activation of proteins in the menisci in the diseased OA compartment, both catabolic and anabolic.

## Author contributions

Conception and design: EF, PÖ, AT and MEProvision of study materials and tissue preparation: ME, EF, VH, PÖ, JT, NA MS analysis: EF and PÖ Statistical analysis: EF, AT, NA and MR Interpretation of results: All co-authors Drafting of the article: EF Critical revision of the article for important intellectual content: MR, AT, PÖ, VH, JT, NA and ME Final approval of the article: All co-authors.

## Conflict of interest

• AT works as an associate editor (statistics) in Osteoarthritis and Cartilage.
• ME reports grants from The European Research Council (ERC), The Foundation for Research in Rheumatology (FOREUM), The Swedish Research Council, The Alfred Österlund Foundation, The Crafoord Foundation, The Swedish Rheumatology Association, The IngaBritt and Arne Lundberg Research Foundation, The Greta and Johan Kock Foundation, and Governmental Funding of Clinical Research within the National Health Service (ALF)
• Other authors (EF, PÖ, NA, JT, VH, MR) report no conflicts of interst.

## Funding

This project has received funding from the 10.13039/501100000781European Research Council (ERC) under the European Union's Horizon 2020 research and innovation programme (grant agreement #771121) (ME) and the 10.13039/501100014034Foundation for Research in Rheumatology (FOREUM) (ME). The study was also funded by grants from the 10.13039/501100004359Swedish Research Council (2014-2348 ME), the 10.13039/501100005390Alfred Österlund Foundation (ME, PÖ), the 10.13039/501100003173Crafoord Foundation (ME, PÖ), the Anna-Greta Crafoord Foundation (PÖ), the 10.13039/501100007949Swedish Rheumatology Association (ME, PÖ), the 10.13039/501100003849IngaBritt and Arne Lundberg Research Foundation (ME, PÖ, MS-instrument), the Krapperup Foundation (PÖ), the Olle Engkvist Foundation (PÖ), the 10.13039/501100006075Greta and Johan Kock Foundation (PÖ, ME), and Governmental Funding of Clinical Research within the National Health Service (ALF) (ME).

## Role of funding source

The funders had no role in study design, data collection and analysis, decision to publish, or preparation of the manuscript.
