# Supplementary material for: Proteomic comparison of osteoarthritic and reference human menisci using data-independent acquisition mass spectrometry
Source: Osteoarthritis Cartilage. 2020 Aug;28(8):1092–101. doi: 10.1016/j.joca.2020.05.001 (PMC7397514; doi:10.1016/j.joca.2020.05.001)
Supplement: Multimedia component 1 [file mmc1.docx]

**Supplementary material**

**Table S1.**

| **Window** | **m/z range** |
| --- | --- |
| 1 | 350-383 |
| 2 | 382-408 |
| 3 | 407-429 |
| 4 | 428-448 |
| 5 | 447-467 |
| 6 | 466-484 |
| 7 | 483-503 |
| 8 | 502-521 |
| 9 | 520-539 |
| 10 | 538-557 |
| 11 | 556-575 |
| 12 | 574-594 |
| 13 | 593-614 |
| 14 | 613-634 |
| 15 | 633-656 |
| 16 | 655-678 |
| 17 | 677-701 |
| 18 | 700-726 |
| 19 | 725-756 |
| 20 | 755-787 |
| 21 | 786-823 |
| 22 | 822-862 |
| 23 | 861-914 |
| 24 | 913-979 |
| 25 | 978-1077 |
| 26 | 1076-1650 |

**Table S2.** Number of missing values for the proteins with more missing values in references than OA samples (n=49), and in which compartment (medial/lateral) the missing values were manifested. No proteins showed the opposite pattern, with more missing in the OA patients.

| **Entry name** | **Protein accessions** | **Missing in medial or lateral meniscus** | **# missing values references**  **(total n=10)** | **# missing values in OA patients**  **(total n=9)** |
| --- | --- | --- | --- | --- |
| PLIN3 | O60664 | Medial | 8 | 2 |
| WDRM | O75083 | Medial | 7 | 1 |
| PAPS2 | O95340 | Medial | 8 | 1 |
| NET1 | O95631 | Medial | 7 | 1 |
| CAH2 | P00918 | Medial | 8 | 2 |
| HBD | P02042 | Lateral | 8 | 0 |
| B3AT | P02730 | Lateral | 8 | 0 |
| FRIL | P02792 | Medial | 7 | 0 |
| CATA | P04040 | Medial | 8 | 2 |
| LKHA4 | P09960 | Medial | 9 | 1 |
| THIO | P10599 | Medial | 8 | 1 |
| TCTP | P13693 | Medial | 8 | 2 |
| HNRPL | P14866 | Medial | 8 | 2 |
| TCPA | P17987 | Medial | 8 | 1 |
| RL7 | P18124 | Medial | 7 | 2 |
| RL17 | P18621 | Medial | 7 | 2 |
| MK | P21741 | Medial | 7 | 2 |
| SAHH | P23526 | Medial | 8 | 2 |
| RL13 | P26373 | Medial | 9 | 2 |
| MAP4 | P27816 | Medial | 7 | 2 |
| CCN2 | P29279 | Medial | 7 | 1 |
| 1433B | P31946 | Medial | 8 | 2 |
| STIP1 | P31948 | Medial | 8 | 2 |
| SYUA | P37840 | Lateral | 8 | 2 |
| MDHM | P40926 | Medial | 8 | 2 |
| SERPH | P50454 | Medial | 8 | 1 |
| TCPQ | P50990 | Medial | 8 | 2 |
| AK1C2 | P52895 | Medial | 9 | 2 |
| PSA6 | P60900 | Medial | 7 | 2 |
| 1433G | P61981 | Medial | 9 | 1 |
| RS23 | P62266 | Medial | 8 | 2 |
| RS26 | P62854 | Medial | 9 | 2 |
| IF5A1 | P63241 | Medial | 7 | 2 |
| TBB4B | P68371 | Medial | 8 | 2 |
| TSG6 | P98066 | Medial | 7 | 1 |
| PRDX4 | Q13162 | Medial | 8 | 1 |
| CAYP1 | Q13938 | Medial | 8 | 2 |
| CIRBP | Q14011 | Medial | 7 | 1 |
| NID2 | Q14112 | Medial | 7 | 0 |
| POSTN | Q15063 | Medial | 8 | 1 |
| PLEC | Q15149 | Medial | 7 | 1 |
| UAP1 | Q16222 | Medial | 9 | 2 |
| TENXA | Q16473 | Medial | 8 | 2 |
| CSPG4 | Q6UVK1 | Medial | 7 | 2 |
| XYLT1 | Q86Y38 | Medial | 7 | 2 |
| CCDC3 | Q9BQI4 | Lateral | 7 | 0 |
| TPPP3 | Q9BW30 | Medial | 8 | 2 |
| MRC2 | Q9UBG0 | Medial | 8 | 2 |
| DKK3 | Q9UBP4 | Medial | 9 | 0 |

**Table S3.** Difference in mean log_2_ intensity differences between the sample groups, here presented as estimates (diff) with the upper (CI higher) and lower (CI lower) limits of 95% confidence intervals (adjusted for age and BMI) for the 331 proteins included in the statistical analysis.

|  |  | **Medial^OA^ vs Medial^ref^** | | | **Lateral^ref^ vs Medial^ref^** | | | **Lateral^OA^ vs Medial^OA^** | | | **Lateral^OA^ vs Lateral^ref^** | | |
| --- | --- | --- | --- | --- | --- | --- | --- | --- | --- | --- | --- | --- | --- |
| **Entry names** | **Protein accessions** | **Diff** | **CI lower** | **CI higher** | **Diff** | **CI lower** | **CI higher** | **Diff** | **CI lower** | **CI higher** | **Diff** | **CI lower** | **CI higher** |
| HBA | P69905 | 4.68 | 3.36 | 6.00 | 1.61 | 0.42 | 2.80 | 0.09 | -1.07 | 1.25 | 3.16 | 1.75 | 4.58 |
| TENA | P24821 | 4.61 | 3.32 | 5.90 | 2.49 | 1.38 | 3.61 | -1.59 | -2.75 | -0.43 | 0.53 | -0.85 | 1.91 |
| SMOC1 | Q9H4F8 | 3.54 | 2.23 | 4.86 | 1.00 | -0.15 | 2.15 | -1.15 | -2.31 | 0.01 | 1.39 | 0.01 | 2.77 |
| SIG16 | A6NMB1 | 3.54 | 2.19 | 4.89 | 0.86 | -0.34 | 2.05 | -1.30 | -2.49 | -0.11 | 1.38 | -0.03 | 2.80 |
| FIBB | P02675 | 3.53 | 2.19 | 4.88 | 0.79 | -0.40 | 1.98 | -1.38 | -2.57 | -0.18 | 1.37 | -0.05 | 2.78 |
| AEBP1 | Q8IUX7 | 3.46 | 2.17 | 4.75 | 1.78 | 0.66 | 2.90 | -0.11 | -1.27 | 1.05 | 1.57 | 0.18 | 2.95 |
| PA2GA | P14555 | 3.44 | 2.15 | 4.73 | 1.02 | -0.10 | 2.14 | -0.22 | -1.38 | 0.94 | 2.20 | 0.82 | 3.58 |
| FNDC1 | Q4ZHG4 | 3.25 | 1.93 | 4.57 | 1.87 | 0.68 | 3.06 | -0.40 | -1.56 | 0.76 | 0.98 | -0.44 | 2.39 |
| CLC3A | O75596 | 3.14 | 1.82 | 4.46 | 0.39 | -0.77 | 1.55 | -2.27 | -3.46 | -1.08 | 0.47 | -0.94 | 1.89 |
| FAM3B | P58499 | 2.97 | 1.65 | 4.28 | 1.72 | 0.56 | 2.87 | -2.09 | -3.25 | -0.93 | -0.84 | -2.22 | 0.54 |
| RAN | P62826 | 2.81 | 1.46 | 4.15 | 1.81 | 0.62 | 3.00 | -1.02 | -2.21 | 0.17 | -0.03 | -1.44 | 1.39 |
| FIBA | P02671 | 2.77 | 1.47 | 4.06 | 1.44 | 0.28 | 2.60 | -0.85 | -2.01 | 0.31 | 0.47 | -0.94 | 1.89 |
| CCD80 | Q76M96 | 2.76 | 1.44 | 4.08 | 1.22 | 0.03 | 2.41 | -0.76 | -1.92 | 0.40 | 0.78 | -0.63 | 2.20 |
| LOXL3 | P58215 | 2.74 | 1.45 | 4.04 | 1.38 | 0.22 | 2.54 | -1.13 | -2.29 | 0.03 | 0.23 | -1.18 | 1.65 |
| DPYL2 | Q16555 | 2.64 | 1.35 | 3.93 | 1.94 | 0.82 | 3.06 | -0.91 | -2.07 | 0.25 | -0.20 | -1.58 | 1.18 |
| FHL1 | Q13642 | 2.63 | 1.33 | 3.92 | 1.38 | 0.26 | 2.50 | -1.14 | -2.30 | 0.02 | 0.11 | -1.27 | 1.49 |
| CO6A2 | P12110 | 2.62 | 1.33 | 3.91 | 1.00 | -0.12 | 2.11 | -1.60 | -2.76 | -0.44 | 0.03 | -1.35 | 1.41 |
| CO6A1 | P12109 | 2.61 | 1.32 | 3.90 | 1.36 | 0.24 | 2.48 | -1.52 | -2.68 | -0.36 | -0.27 | -1.65 | 1.11 |
| BLVRB | P30043 | 2.61 | 1.26 | 3.95 | 1.08 | -0.11 | 2.27 | -0.84 | -2.03 | 0.35 | 0.69 | -0.73 | 2.10 |
| DOT1L | Q8TEK3 | 2.53 | 1.24 | 3.82 | 1.52 | 0.40 | 2.64 | -1.68 | -2.84 | -0.52 | -0.67 | -2.05 | 0.71 |
| FIBG | P02679 | 2.50 | 1.18 | 3.82 | 0.42 | -0.73 | 1.57 | -0.74 | -1.90 | 0.42 | 1.34 | -0.04 | 2.72 |
| LMNA | P02545 | 2.49 | 1.20 | 3.78 | 1.97 | 0.85 | 3.09 | -0.82 | -1.98 | 0.34 | -0.30 | -1.68 | 1.08 |
| MAMC2 | Q7Z304 | 2.48 | 1.19 | 3.77 | 2.81 | 1.69 | 3.93 | -0.18 | -1.34 | 0.98 | -0.51 | -1.89 | 0.87 |
| FINC | P02751 | 2.48 | 1.19 | 3.77 | 0.44 | -0.68 | 1.56 | -1.37 | -2.53 | -0.21 | 0.67 | -0.71 | 2.05 |
| LTBP2 | Q14767 | 2.43 | 1.14 | 3.72 | 2.37 | 1.25 | 3.49 | 1.04 | -0.12 | 2.20 | 1.10 | -0.28 | 2.48 |
| IQGA1 | P46940 | 2.42 | 1.13 | 3.71 | 1.38 | 0.22 | 2.55 | -1.75 | -2.91 | -0.59 | -0.71 | -2.13 | 0.70 |
| IBP5 | P24593 | 2.41 | 1.09 | 3.73 | 0.70 | -0.49 | 1.89 | -0.89 | -2.05 | 0.27 | 0.83 | -0.59 | 2.24 |
| CRYAB | P02511 | 2.37 | 1.08 | 3.66 | 1.13 | 0.01 | 2.25 | -0.56 | -1.72 | 0.60 | 0.68 | -0.70 | 2.06 |
| AHNK | Q09666 | 2.35 | 1.06 | 3.64 | 1.82 | 0.70 | 2.94 | -0.95 | -2.11 | 0.21 | -0.42 | -1.80 | 0.97 |
| ANXA1 | P04083 | 2.33 | 1.04 | 3.62 | 1.50 | 0.38 | 2.62 | -0.87 | -2.03 | 0.29 | -0.04 | -1.43 | 1.34 |
| CD44 | P16070 | 2.32 | 0.97 | 3.66 | 0.82 | -0.37 | 2.01 | -1.15 | -2.34 | 0.04 | 0.35 | -1.07 | 1.76 |
| EF2 | P13639 | 2.30 | 1.01 | 3.59 | 1.46 | 0.30 | 2.62 | -1.23 | -2.39 | -0.07 | -0.39 | -1.81 | 1.02 |
| CO4A2 | P08572 | 2.29 | 0.97 | 3.61 | 1.49 | 0.30 | 2.68 | -1.44 | -2.60 | -0.28 | -0.64 | -2.06 | 0.77 |
| ALDOA | P04075 | 2.27 | 0.97 | 3.56 | 1.66 | 0.54 | 2.77 | -0.69 | -1.85 | 0.47 | -0.08 | -1.46 | 1.30 |
| C1QB | P02746 | 2.26 | 0.94 | 3.58 | 1.84 | 0.68 | 3.00 | -0.98 | -2.17 | 0.21 | -0.57 | -1.98 | 0.85 |
| PRDX2 | P32119 | 2.24 | 0.95 | 3.53 | 1.05 | -0.07 | 2.17 | -0.05 | -1.21 | 1.11 | 1.13 | -0.25 | 2.51 |
| VTDB | P02774 | 2.19 | 0.90 | 3.48 | 1.46 | 0.34 | 2.58 | -0.84 | -2.00 | 0.32 | -0.11 | -1.49 | 1.28 |
| CATD | P07339 | 2.19 | 0.90 | 3.48 | 1.59 | 0.47 | 2.71 | -1.10 | -2.26 | 0.06 | -0.50 | -1.88 | 0.88 |
| ACTBL | Q562R1 | 2.18 | 0.89 | 3.47 | 1.47 | 0.35 | 2.59 | -0.55 | -1.71 | 0.61 | 0.16 | -1.22 | 1.54 |
| PLST | P13797 | 2.18 | 0.86 | 3.50 | 1.96 | 0.80 | 3.12 | -0.75 | -1.94 | 0.45 | -0.53 | -1.94 | 0.89 |
| HNRPM | P52272 | 2.18 | 0.83 | 3.53 | 1.88 | 0.69 | 3.07 | -1.06 | -2.25 | 0.13 | -0.77 | -2.18 | 0.65 |
| ANGI | P03950 | 2.16 | 0.87 | 3.45 | 1.73 | 0.61 | 2.85 | -0.10 | -1.26 | 1.06 | 0.34 | -1.05 | 1.72 |
| IGHA1 | P01876 | 2.16 | 0.87 | 3.45 | 1.17 | 0.05 | 2.29 | 0.17 | -0.99 | 1.33 | 1.16 | -0.22 | 2.54 |
| CSPG2 | P13611 | 2.15 | 0.86 | 3.44 | 1.85 | 0.73 | 2.97 | -0.60 | -1.76 | 0.56 | -0.29 | -1.67 | 1.09 |
| A1BG | P04217 | 2.12 | 0.83 | 3.42 | 0.99 | -0.13 | 2.10 | -0.97 | -2.13 | 0.19 | 0.17 | -1.21 | 1.55 |
| MMP3 | P08254 | 2.11 | 0.82 | 3.41 | 1.60 | 0.44 | 2.76 | -0.07 | -1.23 | 1.09 | 0.44 | -0.97 | 1.86 |
| PTMS | P20962 | 2.11 | 0.79 | 3.43 | 2.00 | 0.85 | 3.15 | -0.26 | -1.42 | 0.90 | -0.15 | -1.53 | 1.23 |
| TPIS | P60174 | 2.08 | 0.79 | 3.37 | 1.61 | 0.49 | 2.73 | -0.46 | -1.62 | 0.70 | 0.01 | -1.37 | 1.40 |
| C1S | P09871 | 2.07 | 0.78 | 3.37 | 1.69 | 0.57 | 2.81 | -0.03 | -1.19 | 1.13 | 0.35 | -1.03 | 1.73 |
| ITIH2 | P19823 | 2.07 | 0.78 | 3.36 | 0.50 | -0.66 | 1.66 | -1.27 | -2.43 | -0.11 | 0.31 | -1.11 | 1.72 |
| PEDF | P36955 | 2.05 | 0.76 | 3.34 | 1.02 | -0.10 | 2.14 | -1.58 | -2.74 | -0.42 | -0.55 | -1.94 | 0.83 |
| ASPN | Q9BXN1 | 2.03 | 0.74 | 3.32 | 0.92 | -0.20 | 2.03 | -1.68 | -2.84 | -0.52 | -0.57 | -1.95 | 0.82 |
| CO6A3 | P12111 | 2.03 | 0.74 | 3.32 | 1.24 | 0.13 | 2.36 | -1.33 | -2.49 | -0.17 | -0.55 | -1.93 | 0.84 |
| BIP | P11021 | 2.02 | 0.73 | 3.31 | 1.24 | 0.12 | 2.36 | -0.48 | -1.64 | 0.68 | 0.30 | -1.08 | 1.68 |
| KPYM | P14618 | 2.02 | 0.73 | 3.31 | 1.33 | 0.22 | 2.45 | -0.78 | -1.94 | 0.38 | -0.09 | -1.48 | 1.29 |
| PPM1M | Q96MI6 | 2.01 | 0.69 | 3.33 | 1.93 | 0.78 | 3.09 | -0.58 | -1.74 | 0.58 | -0.50 | -1.89 | 0.88 |
| EHD2 | Q9NZN4 | 2.01 | 0.72 | 3.30 | 2.00 | 0.84 | 3.16 | -0.78 | -1.94 | 0.38 | -0.77 | -2.18 | 0.65 |
| MYL6 | P60660 | 2.01 | 0.69 | 3.33 | 1.59 | 0.39 | 2.78 | -0.78 | -1.94 | 0.38 | -0.36 | -1.77 | 1.06 |
| PGFRL | Q15198 | 2.00 | 0.71 | 3.29 | 1.05 | -0.11 | 2.22 | -0.59 | -1.75 | 0.57 | 0.36 | -1.06 | 1.77 |
| MOES | P26038 | 2.00 | 0.71 | 3.29 | 1.49 | 0.37 | 2.61 | -0.61 | -1.77 | 0.55 | -0.10 | -1.48 | 1.28 |
| APOE | P02649 | 2.00 | 0.68 | 3.32 | 1.68 | 0.49 | 2.87 | -0.76 | -1.92 | 0.40 | -0.44 | -1.86 | 0.97 |
| KAD1 | P00568 | 1.96 | 0.66 | 3.25 | 1.17 | 0.05 | 2.29 | -0.69 | -1.85 | 0.47 | 0.10 | -1.28 | 1.48 |
| FRIH | P02794 | 1.95 | 0.63 | 3.27 | 1.38 | 0.22 | 2.54 | -0.87 | -2.06 | 0.32 | -0.31 | -1.72 | 1.11 |
| CFAB | P00751 | 1.93 | 0.64 | 3.22 | 0.98 | -0.14 | 2.10 | -1.02 | -2.18 | 0.14 | -0.07 | -1.45 | 1.31 |
| FSCN1 | Q16658 | 1.92 | 0.63 | 3.21 | 1.42 | 0.30 | 2.53 | -0.65 | -1.81 | 0.51 | -0.14 | -1.52 | 1.24 |
| APOA4 | P06727 | 1.92 | 0.63 | 3.21 | 0.19 | -0.93 | 1.31 | -1.16 | -2.32 | 0.00 | 0.56 | -0.82 | 1.94 |
| COFA1 | P39059 | 1.91 | 0.62 | 3.20 | 1.11 | -0.01 | 2.23 | -0.78 | -1.94 | 0.38 | 0.02 | -1.36 | 1.40 |
| LEG1 | P09382 | 1.90 | 0.61 | 3.19 | 1.51 | 0.39 | 2.63 | -0.39 | -1.55 | 0.77 | 0.00 | -1.38 | 1.38 |
| BGH3 | Q15582 | 1.90 | 0.61 | 3.19 | 0.84 | -0.28 | 1.96 | -2.21 | -3.37 | -1.05 | -1.16 | -2.54 | 0.23 |
| PGBM | P98160 | 1.90 | 0.61 | 3.19 | 1.12 | 0.01 | 2.24 | -0.67 | -1.83 | 0.49 | 0.10 | -1.28 | 1.48 |
| TENX | P22105 | 1.85 | 0.56 | 3.14 | 2.00 | 0.88 | 3.12 | -0.58 | -1.74 | 0.58 | -0.73 | -2.11 | 0.65 |
| DAF | P08174 | 1.83 | 0.51 | 3.15 | 1.26 | 0.07 | 2.45 | -0.85 | -2.01 | 0.31 | -0.27 | -1.69 | 1.14 |
| RHOA | P61586 | 1.83 | 0.51 | 3.15 | 1.21 | 0.01 | 2.40 | -0.93 | -2.14 | 0.28 | -0.31 | -1.77 | 1.15 |
| ALBU | P02768 | 1.82 | 0.53 | 3.11 | 1.02 | -0.10 | 2.14 | -0.77 | -1.93 | 0.39 | 0.03 | -1.35 | 1.41 |
| CFAH | P08603 | 1.81 | 0.52 | 3.10 | 1.16 | 0.04 | 2.28 | -0.70 | -1.86 | 0.45 | -0.05 | -1.44 | 1.33 |
| TIMP1 | P01033 | 1.80 | 0.51 | 3.09 | 1.14 | 0.02 | 2.26 | -0.44 | -1.60 | 0.72 | 0.22 | -1.16 | 1.60 |
| PRDX1 | Q06830 | 1.79 | 0.50 | 3.09 | 1.41 | 0.29 | 2.53 | -0.54 | -1.70 | 0.62 | -0.16 | -1.54 | 1.22 |
| ANX11 | P50995 | 1.78 | 0.46 | 3.10 | 1.34 | 0.22 | 2.46 | -0.80 | -1.99 | 0.39 | -0.36 | -1.74 | 1.02 |
| C1QA | P02745 | 1.77 | 0.47 | 3.06 | 1.92 | 0.76 | 3.08 | -0.78 | -1.94 | 0.38 | -0.93 | -2.35 | 0.48 |
| SMOC2 | Q9H3U7 | 1.75 | 0.43 | 3.07 | 0.02 | -1.13 | 1.17 | -0.50 | -1.66 | 0.66 | 1.23 | -0.15 | 2.61 |
| TTHY | P02766 | 1.74 | 0.45 | 3.03 | 0.21 | -0.91 | 1.33 | -0.52 | -1.68 | 0.64 | 1.00 | -0.38 | 2.38 |
| TETN | P05452 | 1.72 | 0.43 | 3.01 | 0.84 | -0.27 | 1.96 | -0.65 | -1.81 | 0.51 | 0.22 | -1.16 | 1.60 |
| SULF2 | Q8IWU5 | 1.71 | 0.42 | 3.00 | 2.01 | 0.85 | 3.17 | -0.20 | -1.36 | 0.96 | -0.50 | -1.92 | 0.91 |
| FETUA | P02765 | 1.70 | 0.41 | 3.00 | 0.97 | -0.15 | 2.09 | -0.85 | -2.01 | 0.31 | -0.12 | -1.50 | 1.27 |
| C1QC | P02747 | 1.68 | 0.39 | 2.97 | 1.59 | 0.43 | 2.75 | -0.52 | -1.68 | 0.64 | -0.43 | -1.84 | 0.99 |
| S10A4 | P26447 | 1.68 | 0.39 | 2.97 | 1.03 | -0.08 | 2.15 | -0.60 | -1.76 | 0.56 | 0.04 | -1.34 | 1.43 |
| ENPL | P14625 | 1.68 | 0.39 | 2.97 | 1.06 | -0.06 | 2.18 | -0.60 | -1.76 | 0.56 | 0.01 | -1.37 | 1.39 |
| HTRA1 | Q92743 | 1.67 | 0.38 | 2.96 | 0.02 | -1.10 | 1.14 | -0.79 | -1.95 | 0.37 | 0.86 | -0.52 | 2.24 |
| K2C1B | Q7Z794 | 1.67 | 0.37 | 2.96 | 1.57 | 0.46 | 2.69 | -0.37 | -1.53 | 0.79 | -0.28 | -1.66 | 1.10 |
| PEBP1 | P30086 | 1.66 | 0.37 | 2.95 | 1.33 | 0.21 | 2.45 | -0.26 | -1.42 | 0.90 | 0.07 | -1.31 | 1.45 |
| K1C9 | P35527 | 1.66 | 0.37 | 2.95 | 2.05 | 0.93 | 3.17 | -0.37 | -1.53 | 0.79 | -0.76 | -2.14 | 0.62 |
| G6PI | P06744 | 1.66 | 0.37 | 2.95 | 1.42 | 0.26 | 2.58 | -0.71 | -1.87 | 0.45 | -0.46 | -1.88 | 0.95 |
| CO8B | P07358 | 1.64 | 0.35 | 2.93 | 0.65 | -0.47 | 1.76 | -1.12 | -2.28 | 0.04 | -0.12 | -1.50 | 1.26 |
| TR11B | O00300 | 1.63 | 0.34 | 2.92 | 0.64 | -0.52 | 1.80 | -0.80 | -1.96 | 0.36 | 0.19 | -1.22 | 1.61 |
| CO5 | P01031 | 1.63 | 0.28 | 2.98 | 0.94 | -0.25 | 2.13 | -1.20 | -2.40 | -0.01 | -0.51 | -1.93 | 0.90 |
| RLA2 | P05387 | 1.62 | 0.28 | 2.97 | 1.07 | -0.08 | 2.22 | -0.33 | -1.52 | 0.86 | 0.22 | -1.16 | 1.60 |
| A1AG2 | P19652 | 1.62 | 0.30 | 2.94 | 1.30 | 0.15 | 2.45 | -0.24 | -1.40 | 0.92 | 0.09 | -1.29 | 1.47 |
| EZRI | P15311 | 1.62 | 0.30 | 2.94 | 1.29 | 0.13 | 2.45 | -0.36 | -1.60 | 0.89 | -0.02 | -1.48 | 1.44 |
| TRFE | P02787 | 1.61 | 0.32 | 2.90 | 0.88 | -0.24 | 2.00 | -0.61 | -1.77 | 0.55 | 0.12 | -1.26 | 1.50 |
| CADH1 | P12830 | 1.59 | 0.24 | 2.93 | 0.79 | -0.40 | 1.98 | -0.62 | -1.81 | 0.57 | 0.18 | -1.24 | 1.59 |
| K1C10 | P13645 | 1.58 | 0.29 | 2.88 | 1.57 | 0.45 | 2.69 | -0.41 | -1.57 | 0.75 | -0.40 | -1.78 | 0.98 |
| CO8A2 | P25067 | 1.58 | 0.28 | 2.87 | 0.88 | -0.24 | 1.99 | -0.20 | -1.36 | 0.96 | 0.50 | -0.88 | 1.88 |
| LV147 | P01700 | 1.57 | 0.25 | 2.89 | 1.13 | -0.03 | 2.28 | -0.21 | -1.37 | 0.95 | 0.23 | -1.15 | 1.62 |
| MGP | P08493 | 1.56 | 0.27 | 2.85 | 0.71 | -0.41 | 1.82 | -0.50 | -1.66 | 0.66 | 0.36 | -1.02 | 1.74 |
| ASSY | P00966 | 1.56 | 0.27 | 2.85 | 1.68 | 0.56 | 2.80 | -0.22 | -1.38 | 0.94 | -0.34 | -1.73 | 1.04 |
| MYOC | Q99972 | 1.56 | 0.24 | 2.88 | 3.48 | 2.36 | 4.60 | -0.17 | -1.36 | 1.02 | -2.09 | -3.48 | -0.71 |
| ANXA4 | P09525 | 1.55 | 0.26 | 2.84 | 1.49 | 0.37 | 2.61 | -0.29 | -1.45 | 0.87 | -0.23 | -1.61 | 1.16 |
| A1AG1 | P02763 | 1.54 | 0.25 | 2.83 | 1.58 | 0.46 | 2.70 | -0.72 | -1.88 | 0.44 | -0.76 | -2.14 | 0.63 |
| CO9 | P02748 | 1.53 | 0.24 | 2.82 | 0.58 | -0.54 | 1.70 | -1.18 | -2.34 | -0.02 | -0.23 | -1.61 | 1.15 |
| PCOC1 | Q15113 | 1.52 | 0.23 | 2.82 | 1.30 | 0.18 | 2.42 | -0.07 | -1.23 | 1.09 | 0.16 | -1.23 | 1.54 |
| RARR2 | Q99969 | 1.50 | 0.21 | 2.79 | 1.04 | -0.08 | 2.16 | -0.39 | -1.55 | 0.77 | 0.07 | -1.31 | 1.46 |
| ROA2 | P22626 | 1.47 | 0.17 | 2.76 | 0.91 | -0.21 | 2.02 | -0.73 | -1.89 | 0.43 | -0.17 | -1.55 | 1.21 |
| PGM1 | P36871 | 1.46 | 0.17 | 2.75 | 1.55 | 0.39 | 2.71 | -0.44 | -1.60 | 0.72 | -0.53 | -1.94 | 0.89 |
| LDHA | P00338 | 1.45 | 0.16 | 2.74 | 1.01 | -0.11 | 2.13 | -0.20 | -1.36 | 0.96 | 0.25 | -1.13 | 1.63 |
| RNAS1 | P07998 | 1.42 | 0.10 | 2.74 | 1.54 | 0.39 | 2.69 | -0.55 | -1.71 | 0.61 | -0.67 | -2.05 | 0.71 |
| ITIH1 | P19827 | 1.42 | 0.13 | 2.71 | 0.85 | -0.27 | 1.97 | -0.48 | -1.64 | 0.68 | 0.09 | -1.29 | 1.47 |
| CPNE3 | O75131 | 1.42 | 0.10 | 2.74 | 1.09 | -0.10 | 2.29 | -0.72 | -1.88 | 0.44 | -0.40 | -1.81 | 1.02 |
| LUM | P51884 | 1.42 | 0.13 | 2.71 | 0.77 | -0.35 | 1.88 | -0.91 | -2.07 | 0.25 | -0.26 | -1.64 | 1.12 |
| H1X | Q92522 | 1.40 | 0.10 | 2.69 | 1.30 | 0.18 | 2.42 | -0.33 | -1.49 | 0.83 | -0.23 | -1.61 | 1.15 |
| HPLN3 | Q96S86 | 1.37 | 0.05 | 2.68 | 2.14 | 1.02 | 3.25 | 0.26 | -0.94 | 1.45 | -0.52 | -1.90 | 0.87 |
| CD59 | P13987 | 1.34 | 0.02 | 2.66 | 1.40 | 0.25 | 2.55 | -0.66 | -1.87 | 0.55 | -0.72 | -2.15 | 0.70 |
| H10 | P07305 | 1.33 | 0.04 | 2.62 | 0.88 | -0.24 | 2.00 | -0.72 | -1.88 | 0.44 | -0.27 | -1.65 | 1.11 |
| H4 | P62805 | 1.30 | 0.01 | 2.59 | 0.37 | -0.75 | 1.49 | -0.60 | -1.76 | 0.56 | 0.33 | -1.05 | 1.71 |
| ZA2G | P25311 | 1.30 | 0.01 | 2.59 | 0.83 | -0.29 | 1.95 | -0.72 | -1.88 | 0.44 | -0.25 | -1.63 | 1.13 |
| NKRF | O15226 | 1.30 | -0.05 | 2.64 | 1.71 | 0.52 | 2.90 | 0.82 | -0.37 | 2.01 | 0.40 | -1.02 | 1.82 |
| LYSC | P61626 | 1.29 | 0.00 | 2.58 | 0.88 | -0.24 | 2.00 | 0.01 | -1.15 | 1.17 | 0.43 | -0.95 | 1.81 |
| KV320 | P01619 | 1.28 | -0.01 | 2.57 | 1.00 | -0.16 | 2.16 | -0.64 | -1.80 | 0.52 | -0.36 | -1.77 | 1.06 |
| TARSH | Q7Z7G0 | 1.28 | -0.01 | 2.57 | 1.29 | 0.18 | 2.41 | -0.28 | -1.44 | 0.88 | -0.29 | -1.67 | 1.09 |
| PROF1 | P07737 | 1.26 | -0.03 | 2.55 | 0.95 | -0.21 | 2.11 | -0.51 | -1.67 | 0.65 | -0.20 | -1.62 | 1.21 |
| RET4 | P02753 | 1.25 | -0.04 | 2.54 | 0.66 | -0.46 | 1.78 | -0.86 | -2.02 | 0.30 | -0.27 | -1.65 | 1.11 |
| HPLN1 | P10915 | 1.25 | -0.05 | 2.54 | 0.36 | -0.76 | 1.48 | -0.86 | -2.02 | 0.30 | 0.02 | -1.36 | 1.40 |
| ANXA2 | P07355 | 1.24 | -0.05 | 2.53 | 0.79 | -0.33 | 1.91 | -0.83 | -1.99 | 0.33 | -0.38 | -1.76 | 1.00 |
| TALDO | P37837 | 1.24 | -0.05 | 2.53 | 1.42 | 0.30 | 2.54 | -0.37 | -1.53 | 0.79 | -0.55 | -1.93 | 0.84 |
| HV307 | P01780 | 1.23 | -0.06 | 2.52 | 0.23 | -0.89 | 1.35 | -0.10 | -1.26 | 1.06 | 0.91 | -0.48 | 2.29 |
| ITIH4 | Q14624 | 1.23 | -0.09 | 2.55 | 0.40 | -0.76 | 1.56 | -0.90 | -2.09 | 0.29 | -0.06 | -1.48 | 1.35 |
| SFRP3 | Q92765 | 1.23 | -0.06 | 2.52 | 1.52 | 0.40 | 2.63 | 0.55 | -0.61 | 1.71 | 0.26 | -1.12 | 1.64 |
| UBA1 | P22314 | 1.23 | -0.06 | 2.52 | 1.42 | 0.26 | 2.58 | -0.44 | -1.60 | 0.72 | -0.64 | -2.05 | 0.78 |
| PLMN | P00747 | 1.21 | -0.08 | 2.50 | 0.76 | -0.36 | 1.88 | -0.34 | -1.50 | 0.82 | 0.12 | -1.26 | 1.50 |
| LDHB | P07195 | 1.20 | -0.15 | 2.55 | 0.83 | -0.36 | 2.03 | 0.05 | -1.14 | 1.24 | 0.41 | -1.00 | 1.83 |
| IGHG3 | P01860 | 1.19 | -0.10 | 2.49 | 0.86 | -0.26 | 1.98 | 0.01 | -1.15 | 1.17 | 0.34 | -1.04 | 1.72 |
| FBLN3 | Q12805 | 1.19 | -0.10 | 2.48 | 0.07 | -1.05 | 1.19 | -1.26 | -2.42 | -0.10 | -0.14 | -1.52 | 1.25 |
| PCOC2 | Q9UKZ9 | 1.19 | -0.10 | 2.48 | 1.17 | 0.05 | 2.29 | -0.30 | -1.46 | 0.86 | -0.29 | -1.67 | 1.09 |
| MYO1C | O00159 | 1.17 | -0.17 | 2.52 | 1.40 | 0.21 | 2.59 | -0.30 | -1.49 | 0.89 | -0.53 | -1.95 | 0.88 |
| NB5R3 | P00387 | 1.16 | -0.16 | 2.48 | 1.16 | -0.03 | 2.35 | -0.42 | -1.58 | 0.74 | -0.41 | -1.83 | 1.00 |
| IGHG2 | P01859 | 1.16 | -0.13 | 2.45 | 0.60 | -0.52 | 1.72 | -0.12 | -1.28 | 1.04 | 0.44 | -0.94 | 1.82 |
| TKT | P29401 | 1.15 | -0.14 | 2.44 | 0.79 | -0.32 | 1.91 | -0.64 | -1.80 | 0.52 | -0.28 | -1.67 | 1.10 |
| RL18 | Q07020 | 1.15 | -0.14 | 2.44 | 0.91 | -0.21 | 2.03 | -0.45 | -1.61 | 0.71 | -0.21 | -1.59 | 1.17 |
| EMIL1 | Q9Y6C2 | 1.14 | -0.15 | 2.43 | 0.88 | -0.24 | 2.00 | -0.19 | -1.35 | 0.97 | 0.07 | -1.31 | 1.45 |
| KCRB | P12277 | 1.14 | -0.21 | 2.49 | 1.10 | -0.09 | 2.29 | -0.61 | -1.80 | 0.59 | -0.57 | -1.98 | 0.85 |
| MIME | P20774 | 1.14 | -0.16 | 2.43 | 0.77 | -0.35 | 1.89 | -0.85 | -2.01 | 0.31 | -0.48 | -1.86 | 0.90 |
| CBR1 | P16152 | 1.13 | -0.16 | 2.42 | 0.77 | -0.35 | 1.89 | -0.10 | -1.26 | 1.06 | 0.27 | -1.11 | 1.65 |
| RL4 | P36578 | 1.13 | -0.19 | 2.45 | 0.57 | -0.62 | 1.76 | -0.33 | -1.49 | 0.83 | 0.23 | -1.18 | 1.65 |
| GELS | P06396 | 1.13 | -0.16 | 2.42 | 1.28 | 0.17 | 2.40 | -0.50 | -1.66 | 0.66 | -0.66 | -2.04 | 0.73 |
| PTGDS | P41222 | 1.09 | -0.20 | 2.38 | 0.18 | -0.93 | 1.30 | -0.11 | -1.32 | 1.11 | 0.80 | -0.63 | 2.22 |
| LAMC1 | P11047 | 1.08 | -0.21 | 2.38 | 1.51 | 0.39 | 2.63 | -0.17 | -1.33 | 0.99 | -0.59 | -1.97 | 0.79 |
| PRDX6 | P30041 | 1.08 | -0.21 | 2.37 | 0.59 | -0.57 | 1.75 | -0.11 | -1.27 | 1.05 | 0.37 | -1.04 | 1.79 |
| PPIB | P23284 | 1.06 | -0.24 | 2.35 | 0.43 | -0.69 | 1.55 | -0.53 | -1.69 | 0.63 | 0.10 | -1.28 | 1.48 |
| ANXA5 | P08758 | 1.04 | -0.25 | 2.33 | 1.37 | 0.25 | 2.48 | -0.25 | -1.41 | 0.91 | -0.57 | -1.95 | 0.81 |
| PDIA1 | P07237 | 1.04 | -0.25 | 2.33 | 0.83 | -0.28 | 1.95 | -0.22 | -1.38 | 0.94 | -0.02 | -1.40 | 1.36 |
| ANGL2 | Q9UKU9 | 1.03 | -0.26 | 2.33 | 0.77 | -0.35 | 1.89 | -1.00 | -2.16 | 0.16 | -0.74 | -2.12 | 0.64 |
| ANT3 | P01008 | 1.02 | -0.27 | 2.32 | 0.72 | -0.40 | 1.84 | -0.53 | -1.69 | 0.63 | -0.22 | -1.61 | 1.16 |
| MXRA5 | Q9NR99 | 1.02 | -0.27 | 2.31 | 0.64 | -0.52 | 1.80 | -0.44 | -1.60 | 0.72 | -0.05 | -1.47 | 1.36 |
| ITIH5 | Q86UX2 | 1.02 | -0.27 | 2.31 | 1.19 | 0.07 | 2.30 | -0.44 | -1.60 | 0.72 | -0.60 | -1.99 | 0.78 |
| CHAD | O15335 | 1.02 | -0.27 | 2.31 | 1.13 | 0.01 | 2.25 | 0.50 | -0.66 | 1.66 | 0.39 | -0.99 | 1.77 |
| IPSP | P05154 | 1.01 | -0.28 | 2.30 | 2.07 | 0.95 | 3.19 | -0.20 | -1.36 | 0.96 | -1.27 | -2.65 | 0.12 |
| TIMP2 | P16035 | 1.00 | -0.29 | 2.29 | 0.21 | -0.95 | 1.37 | -0.98 | -2.14 | 0.18 | -0.19 | -1.61 | 1.22 |
| FA12 | P00748 | 1.00 | -0.30 | 2.29 | 0.50 | -0.61 | 1.62 | -0.79 | -1.95 | 0.37 | -0.29 | -1.68 | 1.09 |
| SRPX2 | O60687 | 1.00 | -0.30 | 2.29 | 0.27 | -0.85 | 1.39 | -1.21 | -2.37 | -0.05 | -0.48 | -1.86 | 0.90 |
| RNAS4 | P34096 | 0.98 | -0.31 | 2.27 | 0.70 | -0.42 | 1.81 | -0.26 | -1.42 | 0.90 | 0.03 | -1.35 | 1.41 |
| OAF | Q86UD1 | 0.97 | -0.32 | 2.26 | 0.39 | -0.77 | 1.55 | 0.07 | -1.09 | 1.23 | 0.65 | -0.76 | 2.07 |
| TIMP3 | P35625 | 0.96 | -0.33 | 2.25 | 0.63 | -0.49 | 1.75 | -0.08 | -1.24 | 1.08 | 0.26 | -1.13 | 1.64 |
| VWA1 | Q6PCB0 | 0.96 | -0.33 | 2.25 | 0.59 | -0.53 | 1.71 | -0.20 | -1.36 | 0.96 | 0.17 | -1.21 | 1.56 |
| APOA1 | P02647 | 0.96 | -0.33 | 2.25 | -0.12 | -1.24 | 1.00 | -0.59 | -1.75 | 0.57 | 0.49 | -0.89 | 1.87 |
| CRAC1 | Q9NQ79 | 0.96 | -0.33 | 2.25 | 0.85 | -0.26 | 1.97 | -0.22 | -1.38 | 0.94 | -0.12 | -1.50 | 1.26 |
| HSPB1 | P04792 | 0.94 | -0.35 | 2.23 | 0.84 | -0.28 | 1.95 | 0.28 | -0.88 | 1.44 | 0.38 | -1.00 | 1.77 |
| AACT | P01011 | 0.94 | -0.35 | 2.23 | 0.68 | -0.44 | 1.80 | -0.55 | -1.71 | 0.61 | -0.29 | -1.67 | 1.10 |
| CO8A | P07357 | 0.94 | -0.35 | 2.23 | 0.30 | -0.82 | 1.42 | -0.71 | -1.87 | 0.45 | -0.07 | -1.45 | 1.32 |
| MFGM | Q08431 | 0.92 | -0.37 | 2.21 | 1.44 | 0.32 | 2.56 | -0.19 | -1.35 | 0.97 | -0.71 | -2.09 | 0.67 |
| CAVN1 | Q6NZI2 | 0.92 | -0.37 | 2.21 | 1.16 | 0.05 | 2.28 | -0.40 | -1.56 | 0.76 | -0.65 | -2.03 | 0.73 |
| ECM2 | O94769 | 0.91 | -0.38 | 2.20 | 0.71 | -0.45 | 1.87 | -0.49 | -1.65 | 0.67 | -0.30 | -1.71 | 1.12 |
| FBN1 | P35555 | 0.91 | -0.38 | 2.20 | 0.60 | -0.52 | 1.72 | -0.56 | -1.72 | 0.60 | -0.25 | -1.63 | 1.13 |
| DEST | P60981 | 0.91 | -0.38 | 2.20 | 0.36 | -0.76 | 1.48 | -0.54 | -1.70 | 0.62 | 0.00 | -1.38 | 1.39 |
| SAMP | P02743 | 0.90 | -0.39 | 2.19 | -0.16 | -1.28 | 0.96 | -0.71 | -1.87 | 0.45 | 0.34 | -1.04 | 1.72 |
| PGS1 | P21810 | 0.90 | -0.40 | 2.19 | 1.04 | -0.08 | 2.16 | -0.15 | -1.31 | 1.01 | -0.30 | -1.68 | 1.08 |
| CILP1 | O75339 | 0.89 | -0.40 | 2.18 | 1.01 | -0.11 | 2.12 | -0.42 | -1.58 | 0.74 | -0.54 | -1.92 | 0.85 |
| PPIA | P62937 | 0.88 | -0.41 | 2.17 | 0.91 | -0.21 | 2.03 | -0.14 | -1.30 | 1.02 | -0.17 | -1.55 | 1.21 |
| FBLN1 | P23142 | 0.88 | -0.41 | 2.17 | 1.52 | 0.41 | 2.64 | 0.68 | -0.48 | 1.84 | 0.04 | -1.34 | 1.42 |
| VTNC | P04004 | 0.87 | -0.42 | 2.16 | 0.06 | -1.06 | 1.17 | -1.49 | -2.65 | -0.33 | -0.68 | -2.06 | 0.70 |
| TAGL2 | P37802 | 0.87 | -0.42 | 2.16 | 0.22 | -0.94 | 1.38 | -1.37 | -2.53 | -0.21 | -0.72 | -2.14 | 0.69 |
| COCA1 | Q99715 | 0.85 | -0.44 | 2.14 | 1.21 | 0.09 | 2.32 | 0.38 | -0.78 | 1.54 | 0.02 | -1.36 | 1.40 |
| RRAS | P10301 | 0.83 | -0.46 | 2.12 | 0.92 | -0.20 | 2.04 | -0.27 | -1.43 | 0.89 | -0.36 | -1.74 | 1.02 |
| HSP7C | P11142 | 0.83 | -0.46 | 2.12 | 0.43 | -0.68 | 1.55 | -0.49 | -1.65 | 0.67 | -0.09 | -1.47 | 1.29 |
| COIA1 | P39060 | 0.82 | -0.47 | 2.11 | 0.78 | -0.33 | 1.90 | -0.64 | -1.80 | 0.52 | -0.60 | -1.98 | 0.79 |
| CD9 | P21926 | 0.81 | -0.48 | 2.11 | 1.27 | 0.15 | 2.39 | -0.02 | -1.18 | 1.14 | -0.47 | -1.85 | 0.91 |
| MFAP2 | P55001 | 0.81 | -0.51 | 2.13 | 0.74 | -0.41 | 1.89 | 0.10 | -1.06 | 1.26 | 0.17 | -1.21 | 1.55 |
| TRY1 | P07477 | 0.81 | -0.48 | 2.10 | 1.23 | 0.11 | 2.34 | -0.20 | -1.36 | 0.96 | -0.62 | -2.00 | 0.76 |
| MATN2 | O00339 | 0.80 | -0.49 | 2.09 | 1.76 | 0.64 | 2.88 | 0.29 | -0.87 | 1.45 | -0.67 | -2.05 | 0.71 |
| CLC11 | Q9Y240 | 0.79 | -0.50 | 2.08 | 0.43 | -0.69 | 1.55 | 0.18 | -0.98 | 1.34 | 0.54 | -0.85 | 1.92 |
| PDIA3 | P30101 | 0.78 | -0.51 | 2.07 | 0.49 | -0.63 | 1.61 | -0.59 | -1.75 | 0.57 | -0.30 | -1.68 | 1.08 |
| CYTC | P01034 | 0.78 | -0.54 | 2.10 | 0.76 | -0.43 | 1.95 | -0.42 | -1.58 | 0.74 | -0.40 | -1.82 | 1.01 |
| PGCA | P16112 | 0.76 | -0.53 | 2.05 | 0.32 | -0.80 | 1.44 | 0.04 | -1.12 | 1.20 | 0.48 | -0.90 | 1.86 |
| PGRP2 | Q96PD5 | 0.76 | -0.54 | 2.05 | 0.36 | -0.80 | 1.52 | -0.63 | -1.79 | 0.53 | -0.23 | -1.65 | 1.18 |
| IDHC | O75874 | 0.75 | -0.57 | 2.07 | 0.68 | -0.43 | 1.80 | 0.23 | -0.96 | 1.43 | 0.30 | -1.08 | 1.68 |
| PFKAP | Q01813 | 0.74 | -0.55 | 2.03 | 0.39 | -0.73 | 1.51 | -0.70 | -1.86 | 0.46 | -0.35 | -1.73 | 1.03 |
| FBLN7 | Q53RD9 | 0.73 | -0.56 | 2.02 | 0.72 | -0.44 | 1.88 | 0.20 | -0.96 | 1.36 | 0.21 | -1.21 | 1.62 |
| ACRO | P10323 | 0.71 | -0.58 | 2.01 | 1.21 | 0.09 | 2.33 | 0.04 | -1.12 | 1.20 | -0.46 | -1.84 | 0.92 |
| ITIH6 | Q6UXX5 | 0.71 | -0.60 | 2.03 | 0.35 | -0.77 | 1.47 | -0.58 | -1.78 | 0.61 | -0.22 | -1.60 | 1.16 |
| IGK | P0DOX7 | 0.67 | -0.62 | 1.96 | 0.56 | -0.56 | 1.68 | 0.16 | -1.00 | 1.32 | 0.26 | -1.12 | 1.64 |
| HEP2 | P05546 | 0.66 | -0.63 | 1.95 | 0.69 | -0.47 | 1.85 | -0.15 | -1.31 | 1.01 | -0.18 | -1.60 | 1.23 |
| FGFP2 | Q9BYJ0 | 0.66 | -0.66 | 1.98 | 1.11 | -0.08 | 2.30 | 0.06 | -1.10 | 1.22 | -0.38 | -1.80 | 1.03 |
| CH3L1 | P36222 | 0.64 | -0.68 | 1.95 | 0.38 | -0.81 | 1.57 | 0.58 | -0.58 | 1.74 | 0.83 | -0.58 | 2.25 |
| CHSTE | Q8NCH0 | 0.63 | -0.69 | 1.95 | 0.65 | -0.51 | 1.81 | 0.02 | -1.17 | 1.22 | 0.00 | -1.41 | 1.42 |
| OMD | Q99983 | 0.62 | -0.69 | 1.94 | 0.43 | -0.76 | 1.63 | -0.47 | -1.63 | 0.69 | -0.28 | -1.69 | 1.14 |
| VIME | P08670 | 0.62 | -0.67 | 1.91 | 0.10 | -1.02 | 1.21 | -0.32 | -1.48 | 0.84 | 0.21 | -1.17 | 1.59 |
| PXDC2 | Q6UX71 | 0.61 | -0.71 | 1.93 | 0.69 | -0.47 | 1.85 | -0.03 | -1.23 | 1.16 | -0.12 | -1.53 | 1.30 |
| GDF10 | P55107 | 0.60 | -0.71 | 1.92 | 1.19 | -0.01 | 2.38 | 0.82 | -0.34 | 1.98 | 0.24 | -1.18 | 1.65 |
| CO2 | P06681 | 0.60 | -0.72 | 1.92 | 0.18 | -1.01 | 1.38 | -0.07 | -1.23 | 1.09 | 0.35 | -1.07 | 1.76 |
| KAIN | P29622 | 0.60 | -0.69 | 1.89 | 0.93 | -0.23 | 2.09 | -0.50 | -1.66 | 0.66 | -0.83 | -2.24 | 0.59 |
| ENOA | P06733 | 0.60 | -0.69 | 1.89 | 0.06 | -1.06 | 1.18 | -0.10 | -1.26 | 1.06 | 0.44 | -0.94 | 1.82 |
| A2GL | P02750 | 0.59 | -0.72 | 1.91 | 0.48 | -0.64 | 1.60 | -0.87 | -2.06 | 0.32 | -0.75 | -2.14 | 0.63 |
| PRELP | P51888 | 0.59 | -0.70 | 1.88 | 0.82 | -0.30 | 1.94 | 0.04 | -1.12 | 1.20 | -0.19 | -1.57 | 1.20 |
| KV401 | P06312 | 0.58 | -0.71 | 1.87 | 0.46 | -0.66 | 1.58 | 0.13 | -1.03 | 1.29 | 0.26 | -1.12 | 1.64 |
| COMP | P49747 | 0.57 | -0.72 | 1.86 | 1.38 | 0.26 | 2.50 | 0.35 | -0.81 | 1.51 | -0.46 | -1.84 | 0.92 |
| PA1B2 | P68402 | 0.56 | -0.73 | 1.85 | 0.13 | -1.03 | 1.29 | -0.08 | -1.24 | 1.08 | 0.35 | -1.07 | 1.76 |
| EDIL3 | O43854 | 0.56 | -0.76 | 1.88 | 0.54 | -0.61 | 1.69 | -0.12 | -1.28 | 1.04 | -0.11 | -1.49 | 1.27 |
| PRG4 | Q92954 | 0.54 | -0.75 | 1.83 | 0.07 | -1.05 | 1.19 | -0.72 | -1.88 | 0.44 | -0.24 | -1.63 | 1.14 |
| DERM | Q07507 | 0.54 | -0.75 | 1.83 | 0.65 | -0.47 | 1.77 | -0.23 | -1.39 | 0.93 | -0.34 | -1.72 | 1.04 |
| IC1 | P05155 | 0.54 | -0.75 | 1.83 | 0.54 | -0.62 | 1.70 | 0.38 | -0.78 | 1.54 | 0.38 | -1.04 | 1.79 |
| GANAB | Q14697 | 0.52 | -0.77 | 1.81 | 0.78 | -0.38 | 1.94 | -0.01 | -1.17 | 1.15 | -0.26 | -1.68 | 1.15 |
| CO7 | P10643 | 0.51 | -0.81 | 1.83 | 0.04 | -1.11 | 1.19 | -0.01 | -1.22 | 1.21 | 0.47 | -0.96 | 1.89 |
| SEPP1 | P49908 | 0.50 | -0.82 | 1.82 | 0.38 | -0.81 | 1.57 | -0.66 | -1.82 | 0.50 | -0.53 | -1.95 | 0.88 |
| ZFHX3 | Q15911 | 0.50 | -0.79 | 1.79 | 0.40 | -0.72 | 1.52 | -0.90 | -2.06 | 0.26 | -0.80 | -2.19 | 0.58 |
| MYADM | Q96S97 | 0.50 | -0.79 | 1.79 | 0.67 | -0.45 | 1.79 | -0.05 | -1.21 | 1.11 | -0.23 | -1.61 | 1.15 |
| PGK1 | P00558 | 0.47 | -0.82 | 1.76 | 0.24 | -0.88 | 1.36 | -0.08 | -1.24 | 1.08 | 0.15 | -1.23 | 1.53 |
| TSP3 | P49746 | 0.46 | -0.83 | 1.75 | 1.02 | -0.10 | 2.14 | -0.19 | -1.35 | 0.97 | -0.75 | -2.14 | 0.63 |
| ANGL7 | O43827 | 0.45 | -0.84 | 1.74 | 1.54 | 0.43 | 2.66 | 1.30 | 0.14 | 2.46 | 0.20 | -1.18 | 1.59 |
| CD109 | Q6YHK3 | 0.44 | -0.85 | 1.73 | 0.79 | -0.33 | 1.91 | 0.18 | -0.98 | 1.34 | -0.17 | -1.55 | 1.21 |
| HEMO | P02790 | 0.44 | -0.85 | 1.73 | 0.37 | -0.75 | 1.49 | -0.33 | -1.49 | 0.83 | -0.26 | -1.64 | 1.12 |
| CD47 | Q08722 | 0.44 | -0.88 | 1.76 | 1.12 | -0.07 | 2.32 | -0.11 | -1.27 | 1.05 | -0.79 | -2.21 | 0.62 |
| HRG | P04196 | 0.43 | -0.86 | 1.72 | -0.27 | -1.39 | 0.85 | -0.74 | -1.90 | 0.42 | -0.04 | -1.42 | 1.34 |
| CO3 | P01024 | 0.42 | -0.87 | 1.72 | 0.00 | -1.12 | 1.12 | -0.01 | -1.17 | 1.15 | 0.41 | -0.97 | 1.79 |
| CAPG | P40121 | 0.41 | -0.91 | 1.73 | 0.57 | -0.55 | 1.69 | -0.69 | -1.88 | 0.50 | -0.85 | -2.23 | 0.54 |
| CILP2 | Q8IUL8 | 0.41 | -0.88 | 1.70 | 0.56 | -0.56 | 1.67 | -0.28 | -1.44 | 0.88 | -0.43 | -1.81 | 0.95 |
| IGG1 | P0DOX5 | 0.40 | -0.89 | 1.69 | 0.38 | -0.74 | 1.50 | 0.19 | -0.97 | 1.35 | 0.21 | -1.17 | 1.59 |
| ANXA6 | P08133 | 0.39 | -0.90 | 1.68 | 0.68 | -0.44 | 1.80 | 0.31 | -0.85 | 1.47 | 0.02 | -1.37 | 1.40 |
| GDIR1 | P52565 | 0.36 | -0.93 | 1.65 | 0.41 | -0.70 | 1.53 | -0.43 | -1.59 | 0.73 | -0.48 | -1.86 | 0.90 |
| APOD | P05090 | 0.36 | -0.93 | 1.65 | 2.06 | 0.90 | 3.22 | 0.42 | -0.74 | 1.58 | -1.29 | -2.70 | 0.13 |
| SODE | P08294 | 0.36 | -0.93 | 1.65 | 1.80 | 0.68 | 2.91 | 0.47 | -0.69 | 1.63 | -0.97 | -2.35 | 0.41 |
| KNG1 | P01042 | 0.34 | -0.95 | 1.63 | -0.02 | -1.14 | 1.09 | -0.22 | -1.38 | 0.94 | 0.15 | -1.23 | 1.53 |
| CAZA2 | P47755 | 0.33 | -0.96 | 1.62 | 0.75 | -0.36 | 1.87 | -0.19 | -1.35 | 0.97 | -0.61 | -1.99 | 0.77 |
| MIA | Q16674 | 0.33 | -0.99 | 1.65 | 0.08 | -1.08 | 1.24 | -0.61 | -1.81 | 0.58 | -0.37 | -1.78 | 1.05 |
| PDIA6 | Q15084 | 0.32 | -1.00 | 1.63 | 0.05 | -1.10 | 1.21 | -0.59 | -1.75 | 0.57 | -0.33 | -1.71 | 1.05 |
| COGA1 | Q07092 | 0.31 | -0.98 | 1.60 | 1.01 | -0.11 | 2.13 | 0.30 | -0.86 | 1.46 | -0.41 | -1.79 | 0.98 |
| ARPC4 | P59998 | 0.30 | -1.02 | 1.62 | 0.44 | -0.75 | 1.63 | 0.12 | -1.10 | 1.33 | -0.02 | -1.48 | 1.44 |
| CFAI | P05156 | 0.30 | -1.02 | 1.62 | 0.12 | -1.03 | 1.27 | -0.46 | -1.62 | 0.70 | -0.28 | -1.66 | 1.10 |
| ANGT | P01019 | 0.30 | -0.99 | 1.59 | 0.43 | -0.73 | 1.59 | -0.10 | -1.26 | 1.06 | -0.22 | -1.64 | 1.19 |
| K2C1 | P04264 | 0.29 | -1.00 | 1.58 | 0.86 | -0.26 | 1.97 | -0.19 | -1.35 | 0.97 | -0.76 | -2.14 | 0.62 |
| COBA2 | P13942 | 0.29 | -1.00 | 1.58 | 0.15 | -0.97 | 1.27 | -0.70 | -1.86 | 0.46 | -0.56 | -1.95 | 0.82 |
| APOH | P02749 | 0.29 | -1.00 | 1.58 | 0.20 | -0.92 | 1.32 | -0.61 | -1.77 | 0.55 | -0.53 | -1.91 | 0.85 |
| CBPB2 | Q96IY4 | 0.27 | -1.02 | 1.56 | 0.63 | -0.53 | 1.79 | -0.48 | -1.64 | 0.68 | -0.83 | -2.24 | 0.59 |
| CLUS | P10909 | 0.25 | -1.04 | 1.54 | 0.27 | -0.85 | 1.39 | 0.02 | -1.14 | 1.18 | 0.01 | -1.37 | 1.39 |
| A1AT | P01009 | 0.25 | -1.04 | 1.54 | 0.23 | -0.88 | 1.35 | -0.19 | -1.35 | 0.97 | -0.17 | -1.56 | 1.21 |
| PPIC | P45877 | 0.24 | -1.10 | 1.59 | -0.15 | -1.34 | 1.04 | -0.08 | -1.27 | 1.11 | 0.32 | -1.10 | 1.73 |
| TSP4 | P35443 | 0.22 | -1.07 | 1.51 | 1.28 | 0.16 | 2.40 | 1.43 | 0.27 | 2.59 | 0.37 | -1.02 | 1.75 |
| CO8A1 | P27658 | 0.22 | -1.07 | 1.51 | 0.19 | -0.93 | 1.31 | -0.70 | -1.86 | 0.46 | -0.67 | -2.05 | 0.71 |
| IBP6 | P24592 | 0.19 | -1.10 | 1.48 | 1.27 | 0.15 | 2.39 | 0.14 | -1.02 | 1.30 | -0.94 | -2.32 | 0.44 |
| FMOD | Q06828 | 0.18 | -1.11 | 1.47 | 1.04 | -0.08 | 2.16 | 0.15 | -1.01 | 1.31 | -0.71 | -2.10 | 0.67 |
| HSP72 | P54652 | 0.16 | -1.13 | 1.46 | 0.25 | -0.87 | 1.36 | -0.13 | -1.29 | 1.03 | -0.21 | -1.59 | 1.17 |
| CO8G | P07360 | 0.15 | -1.14 | 1.44 | -0.14 | -1.26 | 0.98 | -0.64 | -1.80 | 0.52 | -0.35 | -1.73 | 1.03 |
| THRB | P00734 | 0.13 | -1.16 | 1.42 | 0.04 | -1.08 | 1.16 | -0.59 | -1.75 | 0.57 | -0.50 | -1.88 | 0.88 |
| HNRPK | P61978 | 0.04 | -1.27 | 1.36 | 0.37 | -0.78 | 1.52 | 0.07 | -1.09 | 1.23 | -0.25 | -1.63 | 1.13 |
| HP1B3 | Q5SSJ5 | 0.04 | -1.25 | 1.33 | 0.63 | -0.49 | 1.74 | -0.02 | -1.18 | 1.14 | -0.60 | -1.99 | 0.78 |
| GDIB | P50395 | 0.03 | -1.26 | 1.32 | 0.10 | -1.02 | 1.21 | 0.08 | -1.08 | 1.24 | 0.01 | -1.37 | 1.39 |
| CO2A1 | P02458 | 0.03 | -1.26 | 1.32 | -0.76 | -1.88 | 0.36 | -1.11 | -2.27 | 0.05 | -0.32 | -1.70 | 1.06 |
| ANGL5 | Q86XS5 | 0.02 | -1.30 | 1.33 | 0.53 | -0.59 | 1.65 | 0.15 | -1.04 | 1.34 | -0.37 | -1.75 | 1.01 |
| CO4B | P0C0L5 | 0.01 | -1.31 | 1.33 | -0.74 | -1.93 | 0.45 | -0.68 | -1.84 | 0.48 | 0.08 | -1.34 | 1.49 |
| K22E | P35908 | 0.01 | -1.28 | 1.30 | 0.77 | -0.34 | 1.89 | 0.85 | -0.31 | 2.01 | 0.08 | -1.30 | 1.47 |
| CFAD | P00746 | 0.01 | -1.28 | 1.30 | 0.49 | -0.62 | 1.61 | -0.30 | -1.46 | 0.86 | -0.78 | -2.16 | 0.60 |
| OLFL3 | Q9NRN5 | 0.00 | -1.30 | 1.29 | 0.54 | -0.58 | 1.66 | 0.28 | -0.88 | 1.44 | -0.26 | -1.65 | 1.12 |
| GLT10 | Q86SR1 | -0.05 | -1.34 | 1.24 | -0.24 | -1.35 | 0.88 | -1.06 | -2.22 | 0.10 | -0.88 | -2.26 | 0.50 |
| G3P | P04406 | -0.06 | -1.35 | 1.23 | 0.00 | -1.11 | 1.12 | -0.12 | -1.28 | 1.04 | -0.19 | -1.57 | 1.19 |
| TRY3 | P35030 | -0.08 | -1.37 | 1.21 | 0.38 | -0.74 | 1.50 | -0.63 | -1.79 | 0.53 | -1.09 | -2.47 | 0.29 |
| CLIC1 | O00299 | -0.11 | -1.40 | 1.18 | -0.27 | -1.39 | 0.85 | -0.66 | -1.82 | 0.50 | -0.50 | -1.88 | 0.88 |
| GSTP1 | P09211 | -0.11 | -1.40 | 1.18 | -0.15 | -1.27 | 0.97 | 0.16 | -1.00 | 1.32 | 0.21 | -1.17 | 1.59 |
| COBA1 | P12107 | -0.14 | -1.43 | 1.15 | -0.34 | -1.46 | 0.78 | -0.93 | -2.09 | 0.23 | -0.73 | -2.11 | 0.65 |
| EMIL3 | Q9NT22 | -0.15 | -1.44 | 1.14 | 0.37 | -0.75 | 1.48 | 0.07 | -1.09 | 1.23 | -0.44 | -1.83 | 0.94 |
| CERU | P00450 | -0.21 | -1.50 | 1.09 | -0.36 | -1.48 | 0.75 | -0.12 | -1.28 | 1.04 | 0.04 | -1.34 | 1.43 |
| LRP1 | Q07954 | -0.21 | -1.50 | 1.09 | 0.07 | -1.05 | 1.19 | 0.16 | -1.00 | 1.32 | -0.11 | -1.50 | 1.27 |
| SODC | P00441 | -0.22 | -1.51 | 1.08 | 0.24 | -0.88 | 1.36 | 0.16 | -1.00 | 1.32 | -0.29 | -1.67 | 1.09 |
| QSOX1 | O00391 | -0.22 | -1.54 | 1.10 | 0.14 | -1.05 | 1.33 | -0.08 | -1.24 | 1.08 | -0.44 | -1.86 | 0.97 |
| ALDOC | P09972 | -0.25 | -1.54 | 1.04 | 0.08 | -1.04 | 1.20 | 0.19 | -0.97 | 1.35 | -0.14 | -1.52 | 1.24 |
| LYOX | P28300 | -0.34 | -1.63 | 0.95 | 0.23 | -0.88 | 1.35 | -0.37 | -1.53 | 0.79 | -0.95 | -2.33 | 0.43 |
| CO4A5 | P29400 | -0.36 | -1.65 | 0.93 | -0.55 | -1.67 | 0.56 | -0.38 | -1.54 | 0.78 | -0.19 | -1.57 | 1.19 |
| PRR12 | Q9ULL5 | -0.38 | -1.67 | 0.91 | 0.11 | -1.01 | 1.23 | 0.40 | -0.76 | 1.56 | -0.08 | -1.46 | 1.30 |
| RLA0 | P05388 | -0.38 | -1.70 | 0.94 | 0.14 | -0.98 | 1.26 | 0.25 | -0.99 | 1.49 | -0.27 | -1.70 | 1.15 |
| HIPL2 | Q6UWX4 | -0.39 | -1.71 | 0.93 | 0.02 | -1.13 | 1.17 | 0.43 | -0.73 | 1.59 | 0.01 | -1.37 | 1.39 |
| MMP2 | P08253 | -0.42 | -1.71 | 0.87 | -1.02 | -2.14 | 0.10 | -1.36 | -2.52 | -0.20 | -0.76 | -2.14 | 0.62 |
| ADIPO | Q15848 | -0.45 | -1.74 | 0.84 | -0.43 | -1.55 | 0.69 | -0.45 | -1.61 | 0.71 | -0.47 | -1.85 | 0.91 |
| IGKC | P01834 | -0.46 | -1.76 | 0.83 | -0.15 | -1.27 | 0.97 | 0.33 | -0.83 | 1.49 | 0.02 | -1.37 | 1.40 |
| FBX2 | Q9UK22 | -0.52 | -1.81 | 0.78 | 0.01 | -1.15 | 1.17 | 0.41 | -0.75 | 1.57 | -0.12 | -1.53 | 1.30 |
| SPB6 | P35237 | -0.53 | -1.85 | 0.79 | -0.17 | -1.33 | 1.00 | 0.08 | -1.12 | 1.27 | -0.29 | -1.70 | 1.13 |
| CONA1 | Q86Y22 | -0.54 | -1.86 | 0.78 | -0.63 | -1.79 | 0.52 | -0.66 | -1.82 | 0.50 | -0.56 | -1.94 | 0.82 |
| RRP1 | P56182 | -0.58 | -1.90 | 0.74 | -0.92 | -2.07 | 0.23 | -2.31 | -3.47 | -1.15 | -1.96 | -3.34 | -0.58 |
| COSA1 | Q2UY09 | -0.61 | -1.90 | 0.68 | 0.06 | -1.06 | 1.18 | 0.32 | -0.84 | 1.48 | -0.35 | -1.73 | 1.03 |
| GPX3 | P22352 | -0.65 | -1.94 | 0.64 | 0.04 | -1.07 | 1.16 | 0.29 | -0.87 | 1.45 | -0.40 | -1.78 | 0.98 |
| LBP | P18428 | -0.69 | -1.98 | 0.60 | -0.12 | -1.24 | 1.00 | 0.03 | -1.13 | 1.19 | -0.53 | -1.91 | 0.85 |
| KMT2D | O14686 | -0.75 | -2.04 | 0.54 | -0.12 | -1.24 | 1.00 | 0.09 | -1.07 | 1.25 | -0.54 | -1.92 | 0.84 |
| CO1A2 | P08123 | -0.79 | -2.08 | 0.50 | -0.33 | -1.45 | 0.79 | 0.18 | -0.98 | 1.34 | -0.28 | -1.66 | 1.10 |
| CO5A2 | P05997 | -0.82 | -2.11 | 0.47 | -0.58 | -1.70 | 0.54 | -0.28 | -1.44 | 0.88 | -0.52 | -1.90 | 0.87 |
| CO1A1 | P02452 | -0.86 | -2.15 | 0.43 | -0.26 | -1.38 | 0.86 | 0.44 | -0.72 | 1.60 | -0.16 | -1.54 | 1.22 |
| CO3A1 | P02461 | -0.91 | -2.20 | 0.38 | -1.08 | -2.20 | 0.04 | -0.74 | -1.90 | 0.42 | -0.57 | -1.95 | 0.81 |
| AMBP | P02760 | -0.91 | -2.20 | 0.38 | -0.07 | -1.19 | 1.05 | -0.60 | -1.76 | 0.56 | -1.44 | -2.82 | -0.06 |
| PJA1 | Q8NG27 | -0.96 | -2.25 | 0.33 | -0.76 | -1.88 | 0.35 | -0.06 | -1.22 | 1.10 | -0.25 | -1.64 | 1.13 |
| PGS2 | P07585 | -0.98 | -2.27 | 0.32 | -0.31 | -1.43 | 0.81 | -0.22 | -1.38 | 0.94 | -0.88 | -2.26 | 0.50 |
| OTP | Q5XKR4 | -0.98 | -2.27 | 0.31 | -0.79 | -1.91 | 0.32 | 0.47 | -0.69 | 1.63 | 0.28 | -1.10 | 1.66 |
| CO4A4 | P53420 | -1.04 | -2.33 | 0.25 | -0.25 | -1.37 | 0.87 | 0.72 | -0.44 | 1.88 | -0.08 | -1.46 | 1.30 |
| PRB4 | P10163 | -1.04 | -2.33 | 0.25 | -0.10 | -1.21 | 1.02 | 0.11 | -1.05 | 1.27 | -0.84 | -2.22 | 0.54 |
| SATT | P43007 | -1.05 | -2.37 | 0.27 | -0.54 | -1.66 | 0.58 | -0.08 | -1.27 | 1.11 | -0.58 | -1.96 | 0.80 |
| IGA2 | P0DOX2 | -1.10 | -2.42 | 0.22 | -0.78 | -1.90 | 0.34 | -0.09 | -1.28 | 1.10 | -0.42 | -1.80 | 0.97 |
| PKHG3 | A1L390 | -1.21 | -2.50 | 0.08 | -0.71 | -1.83 | 0.41 | 0.24 | -0.92 | 1.40 | -0.26 | -1.64 | 1.12 |
| ALS | P35858 | -1.23 | -2.55 | 0.09 | -0.84 | -2.00 | 0.32 | 0.90 | -0.34 | 2.14 | 0.51 | -0.95 | 1.97 |
| ARX | Q96QS3 | -1.28 | -2.57 | 0.01 | -0.33 | -1.45 | 0.79 | 0.42 | -0.74 | 1.58 | -0.53 | -1.91 | 0.86 |
| COEA1 | Q05707 | -1.35 | -2.64 | -0.06 | -1.04 | -2.15 | 0.08 | 0.65 | -0.51 | 1.81 | 0.34 | -1.04 | 1.72 |
| PRC2C | Q9Y520 | -1.35 | -2.64 | -0.06 | -0.84 | -1.95 | 0.28 | 0.50 | -0.66 | 1.66 | -0.01 | -1.40 | 1.37 |
| CO5A3 | P25940 | -1.37 | -2.66 | -0.08 | -0.87 | -1.98 | 0.25 | 0.42 | -0.74 | 1.58 | -0.09 | -1.47 | 1.29 |
| C1QT5 | Q9BXJ0 | -1.51 | -2.81 | -0.22 | -0.65 | -1.77 | 0.47 | 0.43 | -0.73 | 1.59 | -0.43 | -1.82 | 0.95 |
| TSP1 | P07996 | -1.61 | -2.90 | -0.32 | 1.14 | 0.02 | 2.26 | 1.15 | -0.01 | 2.31 | -1.60 | -2.98 | -0.22 |
| APOC1 | P02654 | -1.73 | -3.02 | -0.44 | -0.92 | -2.08 | 0.24 | 0.78 | -0.38 | 1.94 | -0.03 | -1.44 | 1.39 |
| CO5A1 | P20908 | -2.09 | -3.38 | -0.80 | -1.27 | -2.39 | -0.16 | 0.06 | -1.10 | 1.22 | -0.75 | -2.14 | 0.63 |
| PNPH | P00491 | -2.85 | -4.14 | -1.56 | -0.99 | -2.11 | 0.13 | 0.14 | -1.02 | 1.30 | -1.72 | -3.10 | -0.33 |
| COJA1 | Q14993 | -3.11 | -4.40 | -1.82 | -1.96 | -3.08 | -0.84 | 0.58 | -0.58 | 1.74 | -0.57 | -1.95 | 0.81 |

**
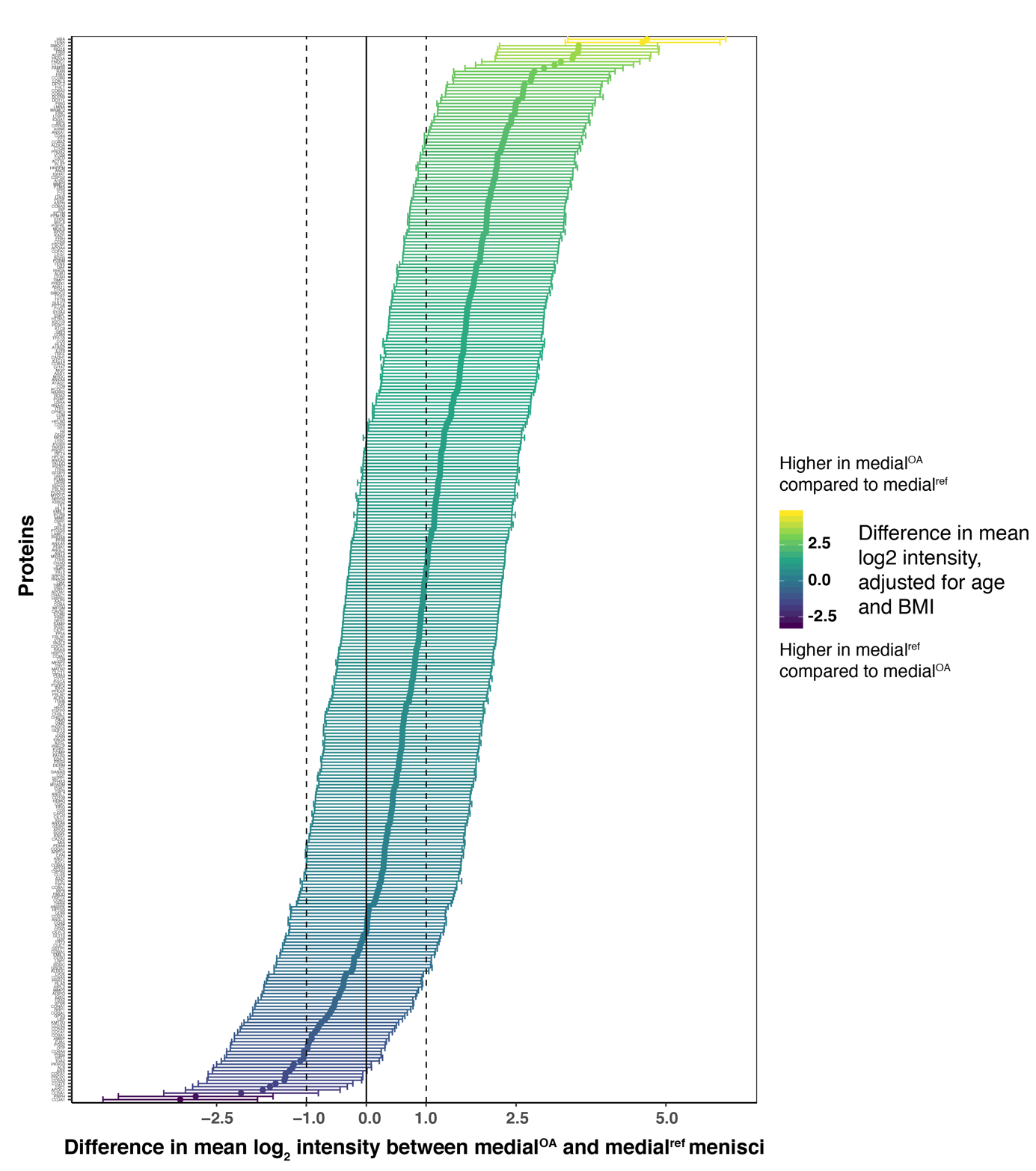
**

**Figure S1.** Visual representation of all 331 proteins included in the statistical analysis and their difference in mean log_2_ intensity between medial^OA^ and medial^ref^ menisci.


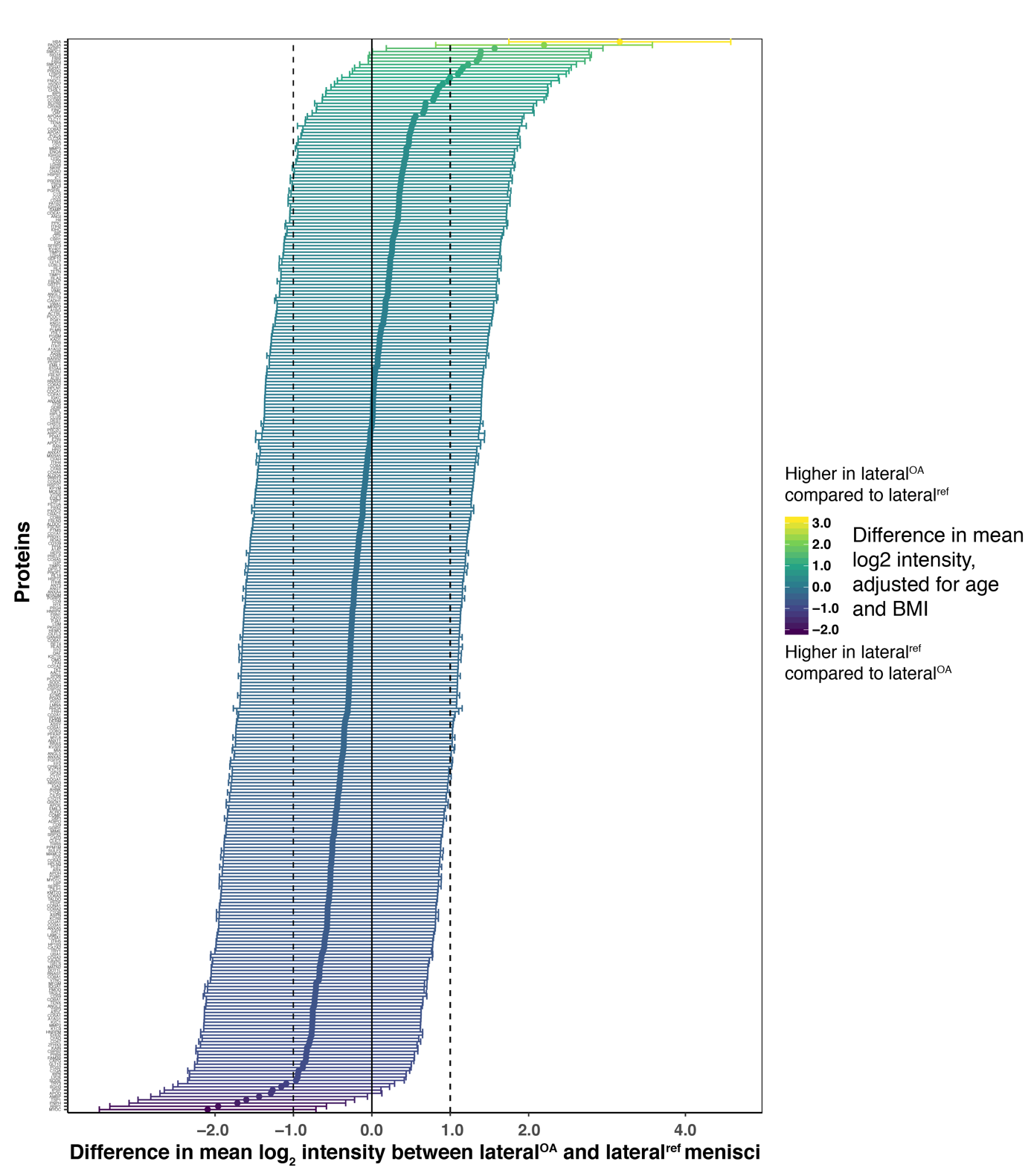


**Figure S2.** Visual representation of all 331 proteins included in the statistical analysis and their difference in mean log_2_ intensity between lateral^OA^ and lateral^ref^ menisci.


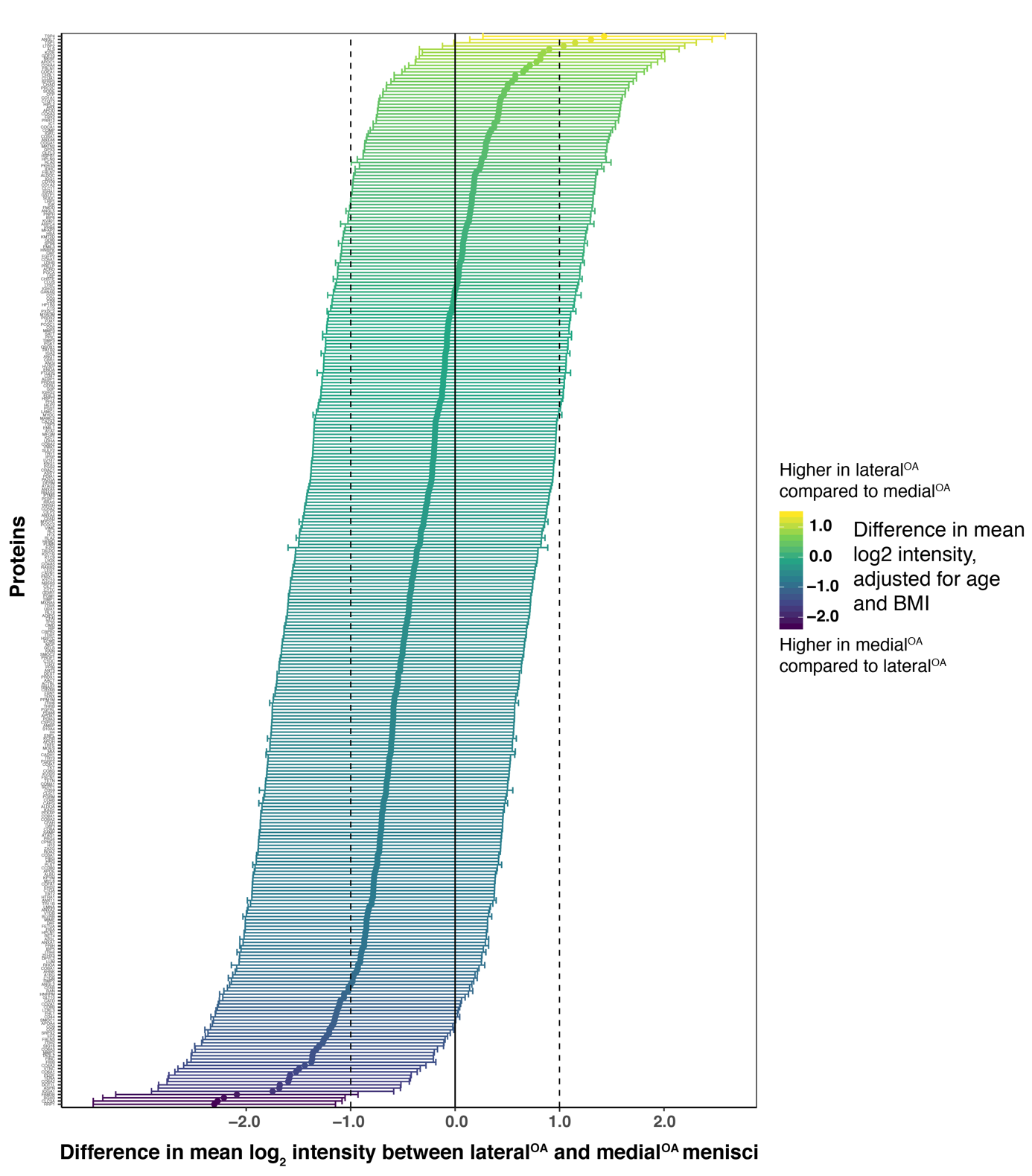


**Figure S3.** Visual representation of all 331 proteins included in the statistical analysis and their difference in mean log_2_ intensity between lateral^OA^ and medial^OA^ menisci.


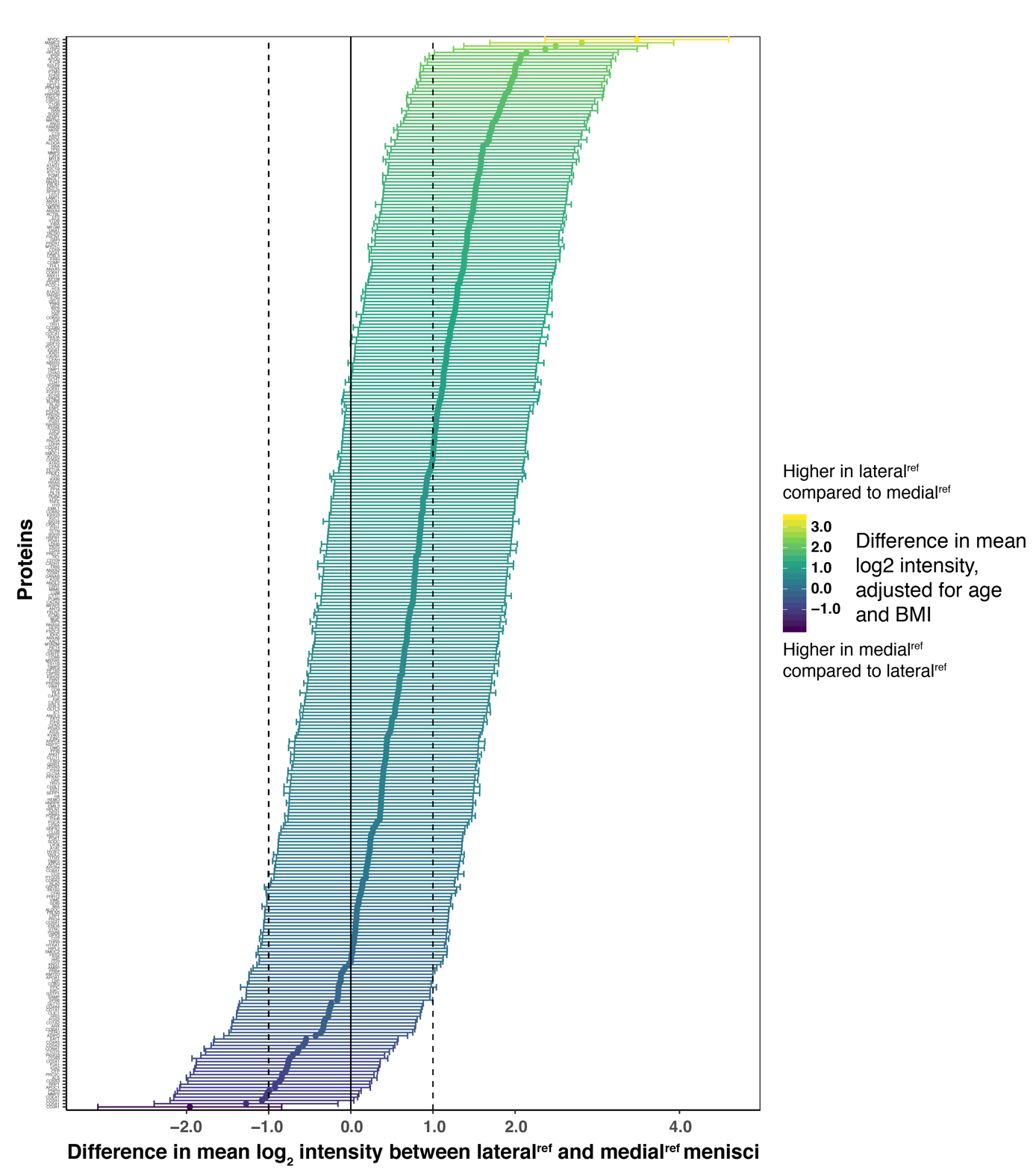


**Figure S4.** Visual representation of all 331 proteins included in the statistical analysis and their difference in mean log_2_ intensity between lateral^ref^ and medial^ref^ menisci.

**Table S4.** Enriched canonical pathways identified in IPA among differentially expressed proteins between medial^OA^ and medial^ref^ menisci.

| **Canonical pathway** | **-log(p-value)** | **z-score** | **Identified proteins mapped to pathway** |
| --- | --- | --- | --- |
| LXR/RXR Activation | 4.01 | 2.83 | A1BG,AHSG,ALB,APOA4,APOC1,APOE,C9,FGA,GC,ITIH4,LYZ,ORM1,ORM2,RBP4,SERPINF1,TF,TNFRSF11B,TTR |
| Production of Nitric Oxide and Reactive Oxygen Species in Macrophages | 1.92 | 2.53 | ALB,APOA4,APOC1,APOE,LYZ,ORM1,ORM2,RBP4,RHOA,TNFRSF11B |
| Signaling by Rho Family GTPases | 0.42 | 2.45 | CDH1,EZR,IQGAP1,MSN,MYL6,RHOA |
| RhoA Signaling | 0.52 | 2.24 | EZR,MSN,MYL6,PFN1,RHOA |
| Role of Pattern Recognition Receptors in Recognition of Bacteria and Viruses | 1.18 | 2.0 | C1QA,C1QB,C1QC,C5 |
| Glycolysis I | 0.90 | 2.0 | ALDOA,GPI,PKM,TPI1 |
| Phospholipase C Signaling | 0.81 | 2.0 | AHNAK,MYL6,PEBP1,PLA2G2A,RHOA |
| Systemic Lupus Erythematosus In T Cell Signaling Pathway | 0.60 | 2.0 | CD44,EZR,MSN,RHOA |
| Coagulation System | 0.47 | 2.0 | FGA,FGB,FGG,PLG |
| ILK Signaling | 0.0 | 2.0 | CDH1,FN1,MYL6,RHOA |
| Leukocyte Extravasation Signaling | 0.95 | 1.89 | CD44,EZR,MMP3,MSN,MYL6,RHOA,TIMP1 |
| Actin Cytoskeleton Signaling | 0.47 | 1.89 | EZR,FN1,IQGAP1,MSN,MYL6,PFN1,RHOA |
| GP6 Signaling Pathway | 1.58 | 1.73 | COL15A1,COL19A1,COL4A2,COL5A1,COL5A3,COL6A1,COL6A2,COL6A3,COL8A2,FGA,FGB,FGG |
| Acute Phase Response Signaling | 3.44 | 1.67 | AHSG,ALB,C1S,C5,C9,CFB,FGA,FGB,FGG,FN1,ITIH2,ITIH4,ORM1,ORM2,PLG,RBP4,SERPINF1,TF,TNFRSF11B,TTR |
| Osteoarthritis Pathway | 1.04 | 1.63 | ANXA2,FN1,FRZB,HTRA1,MMP3,RARRES2,RBP4 |
| Glioma Invasiveness Signaling | 0.69 | 1.0 | CD44,PLG,RHOA,TIMP1 |
| Intrinsic Prothrombin Activation Pathway | 0.41 | 1.0 | COL5A3,FGA,FGB,FGG |
| Sirtuin Signaling Pathway | 0.42 | 0.82 | CDH1,DOT1L,H1F0,H1FX,LDHA,SOD1 |
| Complement System | 2.8 | 0.71 | C1QA,C1QB,C1QC,C1S,C5,C8B,C9,CD55,CD59,CFB,CFH |
| Synaptogenesis Signaling Pathway | 0.0 | 0.0 | APOE,CDH1,RHOA,THBS1 |
| RhoGDI Signaling | 0.57 | -2.45 | CD44,CDH1,EZR,MSN,MYL6,RHOA |
